# ANGPTL2 binds MAG to efficiently enhance oligodendrocyte differentiation

**DOI:** 10.1186/s13578-023-00970-3

**Published:** 2023-02-28

**Authors:** Lu Chen, Zhuo Yu, Li Xie, Xiaoxiao He, Xingmei Mu, Chiqi Chen, Wenqian Yang, Xiaoping Tong, Junling Liu, Zhengliang Gao, Suya Sun, NanJie Xu, Zhigang Lu, Junke Zheng, Yaping Zhang

**Affiliations:** 1 Hongqiao International Institute of Medicine, Shanghai Tongren Hospital/Faculty of Basic Medicine, Key Laboratory of Cell Differentiation and Apoptosis of Chinese Ministry of Education, Shanghai Jiao Tong University School of Medicine, 280 South Chongqing Road, Shanghai, 200025 China; 2 Center for Brain Science, Shanghai Children’s Medical Center, Department of Anatomy and Physiology, Shanghai Jiao Tong University School of Medicine, Shanghai, China; 3 Department of Biochemistry and Molecular Cell Biology, Shanghai Key Laboratory of Tumor Microenvironment and Inflammation, Shanghai Jiao Tong University School of Medicine, Shanghai, China; 4 Yangzhi Rehabilitation Hospital (Shanghai Sunshine Rehabilitation Center), Tongji Univeirsity School of Medicine, Shanghai, China; 5 Department of Neurology and Institute of Neurology, Ruijin Hospital, Shanghai Jiao Tong University School of Medicine, Shanghai, China; 6 Collaborative Innovation Center for Brain Science, Department of Anatomy and Physiology, Key Laboratory of Cell Differentiation and Apoptosis of the Chinese Ministry of Education, Shanghai Key Laboratory of Reproductive Medicine, Shanghai Jiao Tong University School of Medicine, Shanghai, China; 7 The Fifth People’s Hospital of Shanghai, the Shanghai Key Laboratory of Medical Epigenetics, The International Co-Laboratory of Medical Epigenetics and Metabolism, Ministry of Science and Technology, Institutes of Biomedical Sciences, Shanghai Institute of Infectious Diseases and Biosecurity, Shanghai Medical College, Fudan University, Shanghai, China

**Keywords:** ANGPTL2, MAG, Oligodendrocyte, Differentiation, Myelination, Fyn, MYRF

## Abstract

**Background:**

Oligodendrocytes have robust regenerative ability and are key players in remyelination during physiological and pathophysiological states. However, the mechanisms of brain microenvironmental cue in regulation of the differentiation of oligodendrocytes still needs to be further investigated.

**Results:**

We demonstrated that myelin-associated glycoprotein (MAG) was a novel receptor for angiopoietin-like protein 2 (ANGPTL2). The binding of ANGPTL2 to MAG efficiently promoted the differentiation of oligodendrocytes in vitro, as evaluated in an HCN cell line. *Angptl2*-null mice had a markedly impaired myelination capacity in the early stage of oligodendrocyte development. These mice had notably decreased remyelination capacities and enhanced motor disability in a cuprizone-induced demyelinating mouse model, which was similar to the *Mag*-null mice. The loss of remyelination ability in *Angptl2*-null/*Mag*-null mice was similar to the *Angptl2*-WT/*Mag*-null mice, which indicated that the ANGPTL2-mediated oligodendrocyte differentiation effect depended on the MAG receptor. ANGPTL2 bound MAG to enhance its phosphorylation level and recruit Fyn kinase, which increased Fyn phosphorylation levels, followed by the transactivation of myelin regulatory factor (MYRF).

**Conclusion:**

Our study demonstrated an unexpected cross-talk between the environmental protein (ANGPTL2) and its surface receptor (MAG) in the regulation of oligodendrocyte differentiation, which may benefit the treatment of many demyelination disorders, including multiple sclerosis.

**Supplementary Information:**

The online version contains supplementary material available at 10.1186/s13578-023-00970-3.

## Background

Oligodendrocytes are one of the major glial cell types in the central nervous system (CNS) and are essential for myelin formation during CNS development, adaptive myelination in adulthood and remyelination upon damage [1, 2]. However, oligodendrocytes are highly vulnerable and easily affected by trauma, ischemia and immune-mediated demyelinating diseases, such as multiple sclerosis (MS). MS is characterized by focal lymphocytic infiltration, an imbalance between demyelination and remyelination and progressive neurodegeneration [3]. Although much effort was exerted to identify the intrinsic or extrinsic factors involved in the regulation of myelination and remyelination during the activation, migration, proliferation and differentiation of oligodendrocyte progenitors (OPCs) [4, 5, 6, 7, 8, 9, 10], the efficient enhancement of remyelination to delay neurodegeneration remains challenging due to the lack of knowledge on the mechanisms of OPC differentiation into mature oligodendrocytes.

For demyelination-related diseases, the implantation of OPCs into OPC-depleted regions and enhancement of their differentiation into mature and functional oligodendrocytes are two major strategies to promote the remyelination program [11, 12]. Many studies showed that increased numbers of OPCs and some premyelinating oligodendrocytes, but not mature functional oligodendrocytes, predominantly existed in the chronically demyelinated brain area of MS [13, 14, 15], which indicates that full differentiation of oligodendrocytes is critical for remyelination in MS. Although many potential regulators for oligodendrocyte differentiation were reported [16, 17, 18], most of them are intrinsic factors, such as transcription factors (OLIG1, OLIG2, MYRF and SOX10) [19, 20, 21, 22], epigenetic regulators (DNMT1 and DNMT3) [16, 18] and long noncoding RNAs lncOLs [17]. Recently, the importance of extrinsic factors contributing to oligodendrocyte differentiation and their capacity for myelination and remyelination is increasing recognized, such as autotaxin (ATX) [23, 24], Sema3A [25]. However, how extrinsic factors linking receptors to regulating oligodendrocyte maturation remains largely unknown.

The secretory protein angiopoietin-like protein 2 (ANGPTL2) is a member of the angiopoietin-like family that plays vital roles in multiple physiological and pathological states, including angiogenesis [26, 27, 28], lipid metabolism [29], obesity [30], thrombosis [31], atherosclerosis [32] fibrosis [33] and tumor metastasis [34, 35] Our previous study demonstrated that ANGPTL2 was a ligand for the immune-inhibitory receptor human leukocyte immunoglobulin-like receptor B2 (LILRB2), maintained the stemness of hematopoietic stem cells (HSCs) and enhanced leukemogenic activities [36]. Notably, we screened a membrane protein library and identified another receptor, MAG, that bound to ANGPTL2 with high binding affinity (~ 10 nM). ANGPTL2-MAG-mediated signaling promoted the differentiation of oligodendrocytes in vitro and in vivo and enhanced remyelination in a cuprizone-induced demyelination murine model. ANGPTL2 enhanced the phosphorylation of MAG to recruit and activate Fyn-mediated signaling and increase the expression of myelination-related genes, such as the transcription factor, MYRF. These findings provide unique insight into the regulation of the differentiation of oligodendrocytes and a potential strategy for the treatment of demyelination diseases.

## Results

### Human MAG is a new receptor for ANGPTL2

**Fig. 1:**
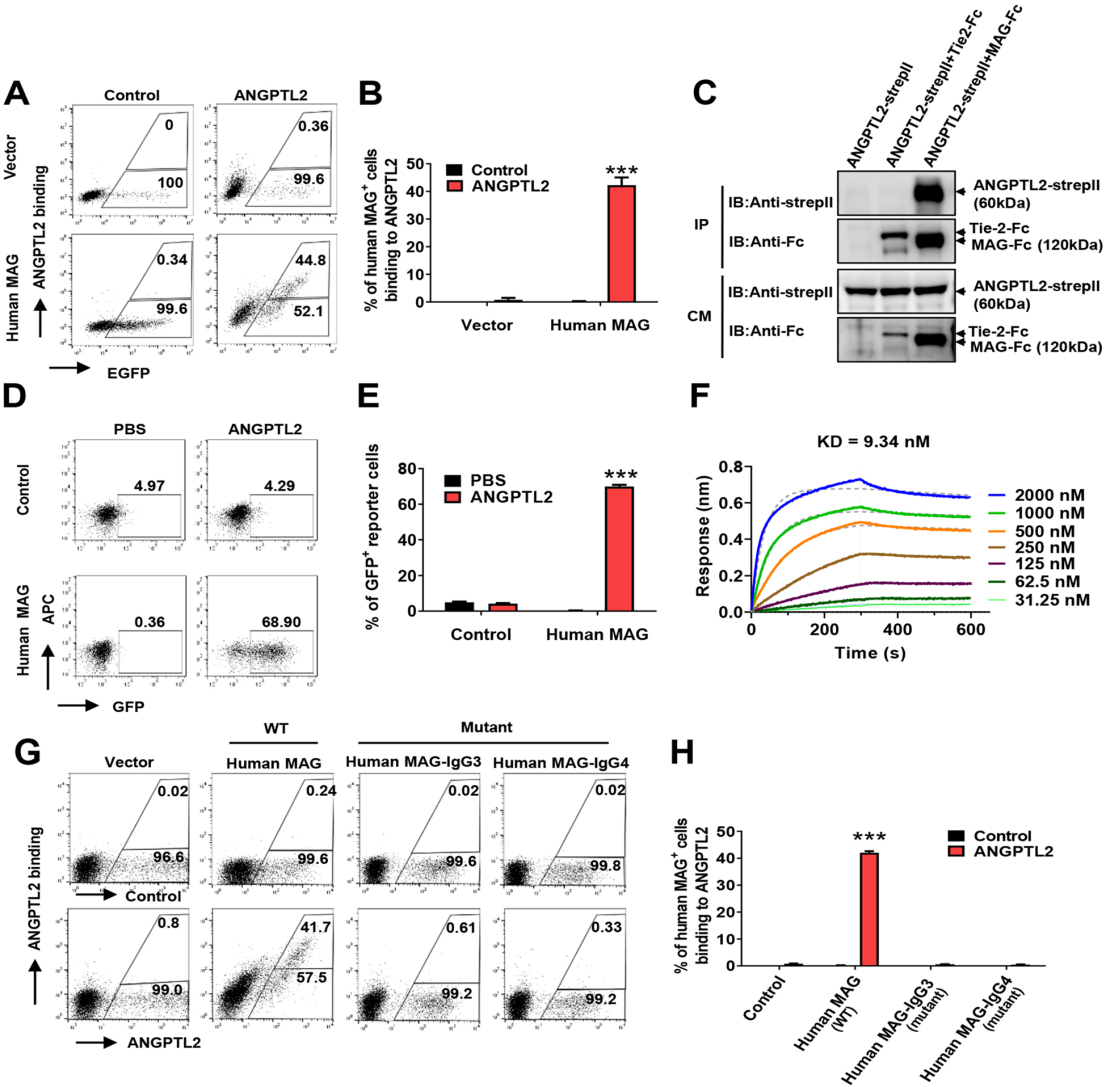
Human MAG is a new receptor for ANGPTL2. **A** Flow cytometric analysis of conditioned medium with/without ANGPTL2-Flag binding to 293T cells with transient transfection of plasmids of N1-hMAG-EGFP (human MAG, full-length) or N1-EGFP (vector). Percentage of binding/unbinding cells to ANGPTL2 in EGFP^+^ cells are shown. **B** Quantitative data of percentage of binding cells in Panel A (n = 3). **C** ANGPLT2 bound to the ECD of human MAG (human MAG-ECD-FC) in the conditioned medium (CM) of cotransfected 293T cells using co-immunoprecipitation. Tie-2-ECD-FC served as a negative control. **D** Representative flow cytometric analysis showing that purified ANGPTL2-Flag protein enhanced GFP expression in human MAG chimeric reporter cells (Human MAG). Control reporter cells (control) without human MAG-ECD were examined. **E** Quantitative data in Panel **D** (n = 3). **F** Binding kinetics of ANGPTL2-Flag to human MAG-ECD were measured using a biolayer interferometry instrument (Octed RED 96/96S). **G**–**H** Flow cytometric plots for the binding of ANGPTL2-Flag to full-length, mutant IgG 3 domain or IgG 4 domain of human MAG expressed 293T cells. Percentage of binding/unbinding cells to ANGPTL2 in EGFP^+^ cells (**G**) and quantitative data are shown (**H**) (n = 3). (***p < 0.001)

We previously showed that ANGPTL2 bound LILRB2 to maintain HSC activities. We further screened other potential surface molecules that may be receptors for ANGPTL2 using flow cytometric analysis and a customized human cDNA library of membrane proteins (Additional file 1: Fig. S1A). Notably, MAG, which is a type I single-pass transmembrane glycoprotein expressed in oligodendrocytes and Schwann cells that plays important roles in myelination in the CNS and peripheral nervous system (PNS) [37, 38] as a ligand for NgR [39], PirB [40] and β1-integrin [41], or as a receptor for gangliosides [42], specifically bound ANGPTL2, but not other ANGPTL members (Fig. 1A–B, Additional file 1: Fig. S1B–C). Immunoprecipitation assays further showed that ANGPTL2 interacted with the extracellular domain (ECD) of MAG (Fig. 1C). A chimeric receptor assay [43] was established to determine the interaction between ANGPTL2 and MAG. The MAG ECD was fused with transmembrane/intracellular domains of activating paired immunoglobulin-like receptor b (PILRb) in this system, followed by infection into murine T-cell hybridoma cells with a nuclear factor of activated T cells (NFAT)/GFP reporter gene (Additional file 1: Fig. S1D). We observed that ANGPTL2 induced marked GFP expression in MAG reporter cells, as determined by flow cytometry (Fig. 1D–E).

We evaluated the binding affinity between ANGPTL2 and MAG using biolayer interferometry (Octet) and surface plasmon resonance (SPR), which showed that the dissociation constants were 9.34 nM (Fig. 1F) and 6.47 nM (Additional file 1: Fig. S1E), respectively. We previously showed that the H*G*Y*C motifs of LILRB2 were essential for its binding to ANGPTL2 [43]. We examined the potential binding motifs of the extracellular IgG domain of MAG and speculated that G301/Y303 and G389/Y341 in the third or fourth IgG domain were the key binding motifs for ANGPTL2. Mutagenesis analysis showed that G301D/Y303A MAG mutation in IgG3 and G389D/Y341A in IgG4 abolished binding to ANGPTL2 than the MAG control, as determined by flow cytometric analysis (Fig. 1G–H), which suggests that IgG3/IgG4 is critical for ANGPTL2-mediated binding/activation of MAG.

### Murine homologue of human MAG binds to ANGPTL2

**Fig. 2:**
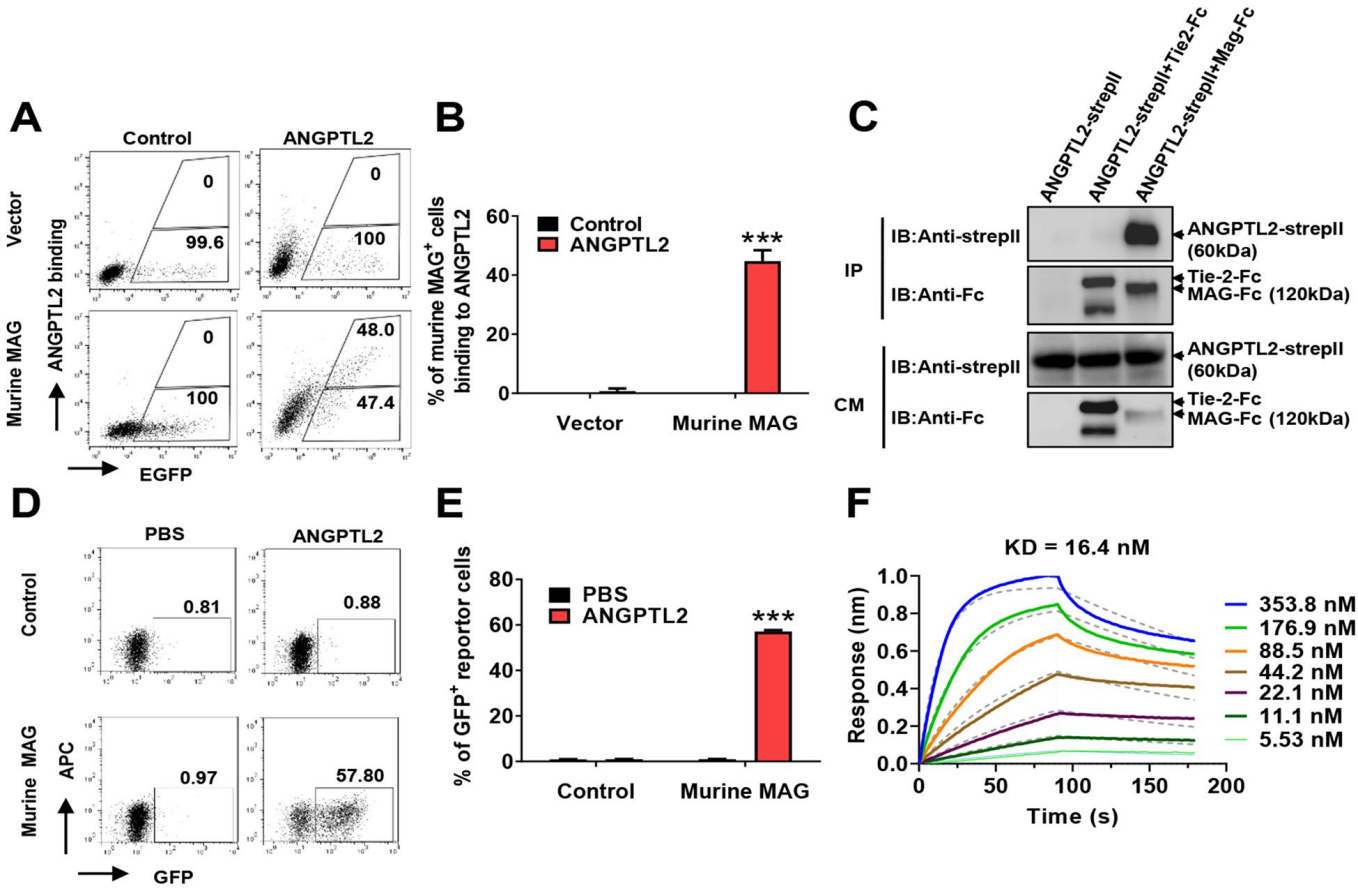
Murine homologue of human MAG binds to ANGPTL2. **A** Flow cytometric analysis of conditioned medium with/without ANGPTL2-Flag binding to 293 T cells with transient transfection of plasmids of N1-mMAG-EGFP (murine MAG, full-length) or N1-EGFP (vector). Percentage of binding/unbinding cells to ANGPTL2 in EGFP^+^ cells are shown. **B** Quantitative data of percentage of binding cells in Panel **A** (n = 3). **C** ANGPLT2 bound to the ECD of murine MAG (murine MAG-ECD) in the conditioned medium (CM) of 293 T cotransfected cells using co-immunoprecipitation. Tie-2-ECD-FC served as a negative control. **D** Representative flow cytometric analysis showing that purified ANGPTL2-Flag protein enhanced GFP expression in murine MAG chimeric reporter cells (Murine MAG). Control reporter cells (control) without murine MAG-ECD were examined. **E** Quantitative data in Panel D (n = 3). **F** Binding kinetics of ANGPTL2-Flag to murine MAG-ECD were measured using a biolayer interferometry instrument (Octed RED 96/96S). (***p < 0.001)

We determined whether ANGPTL2 also bound to the murine homologue of human MAG. Notably, murine MAG exhibited 94% identical sequence to human MAG, which indicates that this gene is very conserved between different species. Similar to human MAG, we found that ANGPTL2 bound murine MAG using flow cytometric analysis (Fig. 2A–B). Co-immunoprecipitation assays showed that ANGPTL2 directly interacted with murine MAG (Fig. 2C). We further constructed chimeric reporter cells with murine MAG ECD and revealed that ANGPTL2 efficiently induced GFP expression in reporter cells (Fig. 2D–E). Notably, the dissociation constant of ANGPTL2 to murine MAG was similar to human MAG as determined by Octet (16.4 nM, Fig. 2F) and SPR (3.08 nM, Additional file 1: Fig. S2A). These data clearly showed that MAG was the receptor for ANGPTL2 with high affinity.

### ANGPTL2 promotes oligodendrocyte differentiation

*Mag* deletion disrupted and multiplied compact myelin lamella and increased periaxonal spacing in the murine CNS [44, 45]. Notably, the newly identified receptor MAG was highly expressed in oligodendrocytes, but not oligodendrocyte precursor cells (https://www.proteinatlas), which indicates that ANGPTL2-MAG mediated signaling may play important roles in oligodendrocyte maturation at late differentiation stage (but not in early precursor cell stage).

To determine whether ANGPTL2-MAG-mediated signaling is required for the oligodendrocyte maturation, we first established an in vitro oligodendrocyte differentiation system using a rat hippocampus-derived adult neural progenitor (HCN) cell line, which efficiently differentiates into mature oligodendrocytes in the presence of IGF1 [46]. HCN cells are efficiently induced into mature oligodendrocytes with small and round somata and web-like branches in morphology. These cells were positive for several markers of mature oligodendrocytes, such as myelin basic protein (MBP) [47], myelin-associated glycoprotein (MAG) [48, 49] and myelin-oligodendrocyte glycoprotein (MOG) [47] (Additional file 1: Fig. S3A), which were increased at the mRNA and protein levels over time (Additional file 1: Fig. S3B–C).

**Fig. 3:**
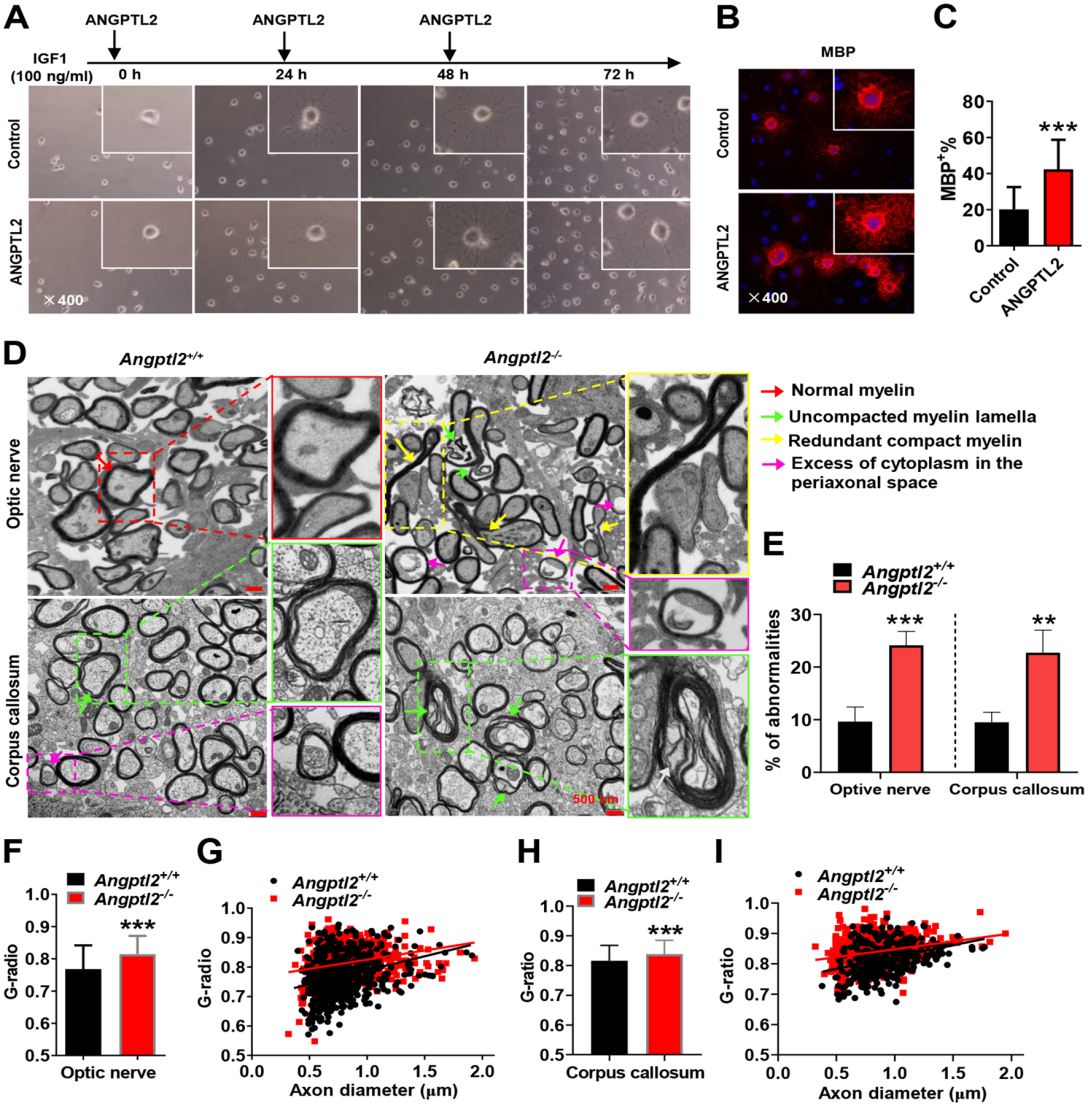
ANGPTL2 promotes oligodendrocyte differentiation. **A** Cell morphology of HCN cells after treatment with IGF1 for the differentiation to oligodendrocytes in vitro with/without ANGPTL2 at the indicated time points. **B** Immunofluorescence staining for MBP in HCN cells 72 h after induction in Panel A. **C** Quantitative data in Panel B are shown; A total of 22 or 23 sections were counted (n = 4). **D** Representative electron microscopy images in the optic nerve fiber and corpus callosum of the brains from *Angptl2*^+*/*+^ and *Angptl2*^−/−^ mice on day 15; red arrow indicates the normal myelin, green arrow indicates uncompacted myelin lamella; yellow arrow indicates redundant compact myelin; purple indicates excess of cytoplasm in the periaxonal space. **E** Shown are the quantification data of abnormally myelinated axons including uncompacted myelin lamella, redundant compact myelin and excess of cytoplasm in the periaxonal space, in optic nerve fibers and corpus callosum. 100–410 axons in each mouse were counted (n = 3). **F**–**I** Quantification of the G ratios of the myelinated axons of the optic nerve fiber (**F**) or corpus callosum (**H**) in D. A total of 120 ~ 200 axons in each mouse were counted (n = 3). The scatter plots for the individual G-ratio values and axonal size distribution in the optic nerve fibers (**G**) or corpus callosum (**I**) in Panel D are shown. (***p < 0.001)

We further treated HCN cells with ANGPTL2 in the presence of IGF1 and demonstrated that more morphological oligodendrocytes with web-like branches were elicited compared to the control (Fig. 3A). Immunofluorescence staining also showed a notable increase in the number of mature MBP^+^ oligodendrocytes with more complicated web-like branches upon ANGPTL2 treatment (20.15% *vs.* 42.36%, Fig. 3B–C), which indicates an accelerated processes of oligodendrocyte maturation. Because we previously showed that ANGPTL2 bound to LILRB2 and its murine ortholog, paired Ig-like receptor (PirB), to maintain the stemness of HSCs [36], we further examined PirB expression during the differentiation from HCN cells in vitro. Notably, the Pirb mRNA level was almost undetectable, but the expression levels of Mag, Mbp, Mog and Angtpl2 were increased during the process of oligodendrocyte differentiation (Additional file 1: Fig. S3D–E), which suggested that MAG was a specific receptor for ANGPTL2 and ANGPTL2 might be secreted by oligodendrocytes to exert its autocrine effect in promoting oligodendrocyte differentiation. Meanwhile, the frequencies of PDGFa^+^Ki-67^+^ oligodendrocyte precursor cells were comparable to the control group, supporting the notion that ANGTPL2 had no effect on the proliferation and differentiation of precursor cells (Additional file 1: Fig. S3F–G).

To further examine the role of ANGPTL2 in oligodendrocyte differentiation, we established *Angptl2* knockout mice with deletion of exon 2 by breeding male *Angptl2*^*fl/fl*^; *Stra8-cre* mice (*Angptl2* was specifically deleted in early-stage spermatogonia, spermatocytes and sperm) with wild-type (WT) female mice (Additional file 1: Fig. S3H). The genetic deletion of *Angptl2* was further confirmed using PCR (Additional file 1: Fig. S3I). No detectable ANGPTL2 protein was observed in different mouse tissues of *Angptl2*-null (or *Angptl2*^*−/−*^) mice, including the heart, lung and brain, as determined by Western blot (Additional file 1: Fig. S3J). Notably, *Angptl2-null* mice had several defects in the myelination of axons, such as uncompacted myelin lamella, redundant compact myelin and excess of cytoplasm in the periaxonal space, in both optic nerves and corpus callosum as evaluated by using transmission electron microscopy (Fig. 3D–E). The thickness of myelin lamella in the optic nerve and corpus callosum from *Angptl2-null* mice was significantly thinner than WT mice at the early neonatal stage (Day 15, Fig. 3F–I), as indicated by an increased G-ratio, but not at the late adult stage (Day 35, Additional file 1: Fig. S3K–N). These results indicate that ANGPTL2 plays a vital role in myelin formation at the early stage of mouse development, which is consistent with the findings in *Mag*-null mice with delayed myelination [44, 45] and further suggest that ANGPTL2-MAG-mediated signaling is crucial for oligodendrocyte differentiation.

### ANGPTL2 promotes oligodendrocyte differentiation and remyelination under pathological conditions

**Fig. 4:**
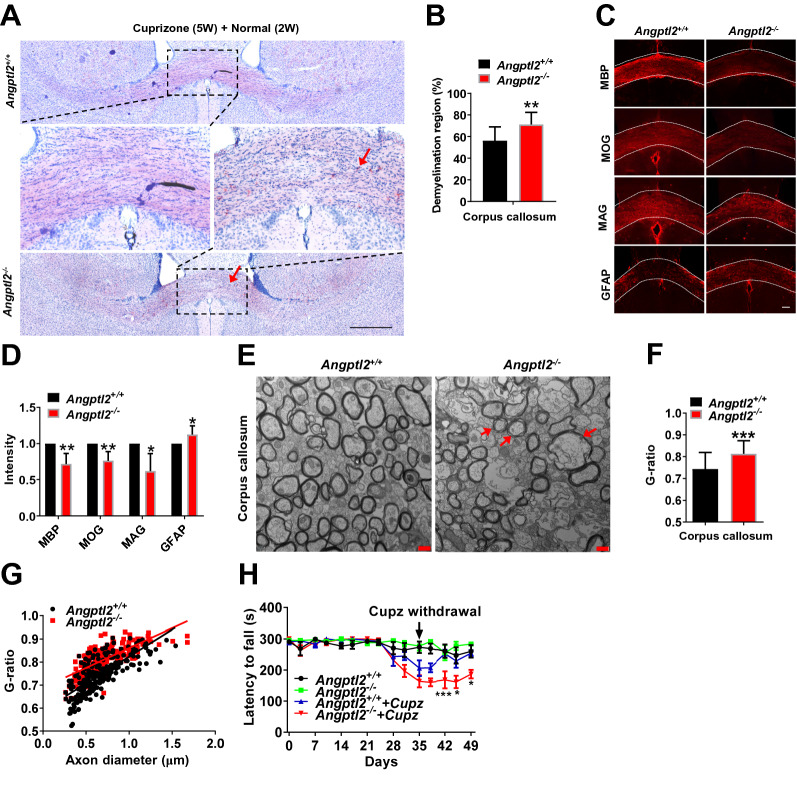
ANGPTL2 promotes oligodendrocyte differentiation and remyelination under pathological conditions. **A** Representative images of the corpus callosum region stained with oil red O in *Angptl2*^+*/*+^ and *Angptl2*^*−/−*^ mice after the withdrawal of cuprizone for two additional weeks. Myelin was stained in red color. Meanwhile, more nonspecific oil red O positive deposits (a phenomenon that oil red O deposits in damaged myelin region) were observed in *Angptl2*^*−/−*^ mice (arrows). **B** Quantitative data of the demyelinating areas in Panel A; six sections from each mouse were analyzed (n = 7). **C** Immunofluorescence staining for MBP, MOG, MAG and GFAP in the corpus callosum region from *Angptl2*^+*/*+^ and *Angptl2*^*−/−*^ mice after the withdrawal of cuprizone for two additional weeks. **D** Quantification of the fluorescence intensity of MBP, MOG, MAG and GFAP in the corpus callosum region in Panel C. Four sections from each mouse were analyzed (n = 6–7). **E** Representative electron microscopy images of the corpus callosum from *Angptl2*^+*/*+^ and *Angptl2*^*−/−*^ mice after the withdrawal of cuprizone for two additional weeks; red arrow indicates uncompacted myelin lamella; **F** Quantification of the G ratios of the remyelinated axons of the corpus callosum in Panel **E**. Approximately 70 axons were counted per mouse (n = 3). **G** The scatter plot for the individual G-ratio values and axonal size distribution of the corpus callosum in Panel E. **H** Motor coordination of *Angptl2*^+*/*+^ and *Angptl2*^*−/−*^ mice after cuprizone (Cupz) treatment for five weeks and cuprizone withdrawal for two additional weeks in the rotarod test; 7–8 mice were used for each group. (*p < 0.05, **p < 0. 01, ***p < 0.001)

To further evaluate the role of ANGPTL2 in pathological states, we used a cuprizone-induced murine demyelination model [50] and examined the dynamic changes in myelination in the corpus callosum. The demyelination status in the corpus callosum was similar between WT and *Angptl2*-null mice 5 weeks after cuprizone diet treatment as determined by oil red O staining (Additional file 1: Fig. S4A–B). Immunohistochemical staining also showed similar decreased levels of several myelin related proteins, including MBP, MOG and MAG (Additional file 1: Fig. S4C–D). The expression level of a marker of astrocyte activation, GFAP, was also comparable between WT and *Angptl2*-null mice (Additional file 1: Fig. S4C–D), although it has been reported to be upregulated in brain due to the compensatory effect from demyelination after cuprizone treatment [51]. These data also indicated that ANGPTL2 had no effect on the process of demyelination. Notably, spontaneous remyelination in the corpus callosum in *Angptl2*^*−/−*^ mice was much slower as evidenced by less myelin staining, while more nonspecific oil red O positive deposits (a phenomenon that oil red O deposits in damaged myelin region [52]), than the WT mice after the withdrawal of cuprizone for an additional 2 weeks, as measured by oil red O staining (Fig. 4A–B). *Angptl2*-null mice also had decreased expression levels for myelin related proteins MBP, MOG and MAG, but increased astrocyte marker of GFAP due to the compensatory effect, as determined by the immunohistochemical staining (Fig. 4C–D).

Although very severe demyelination was observed using electron microscopy 5 weeks after cuprizone treatment (Additional file 1: Fig. S4E), the newly formed myelin in WT mice was more tightly wrapped and integrated than the *Angptl2*-null mice 2 weeks after cuprizone withdrawal (Fig. 4E). The myelin sheath in *Angptl2*-null mice was thinner than the WT mice, as evaluated by the G-ratio of the remyelinated axons (Fig. 4F–G). To test the difference in functional recovery after cuprizone treatment, a widely used assay for the assessment of motor coordination in rodents, the rotarod test, was used in WT and *Angptl2*-null mice to measure motor coordination and balance. The motor of WT and *Angptl2*-null mice began to decline compared to control mice fed normal chow 24 days after cuprizone treatment (Fig. 4H). However, *Angptl2*-null mice had a shorter latency to falling from the rotarod instrument than WT mice (160 s vs. 227.4 s at day 45, 185.2 s vs. 252.8 s at day 49, Fig. 4H) after cuprizone withdrawal, which indicated that ANGPTL2 promoted functional recovery from demyelination.

### ANGPTL2 promotes oligodendrocyte differentiation and remyelination via its receptor MAG

**Fig. 5:**
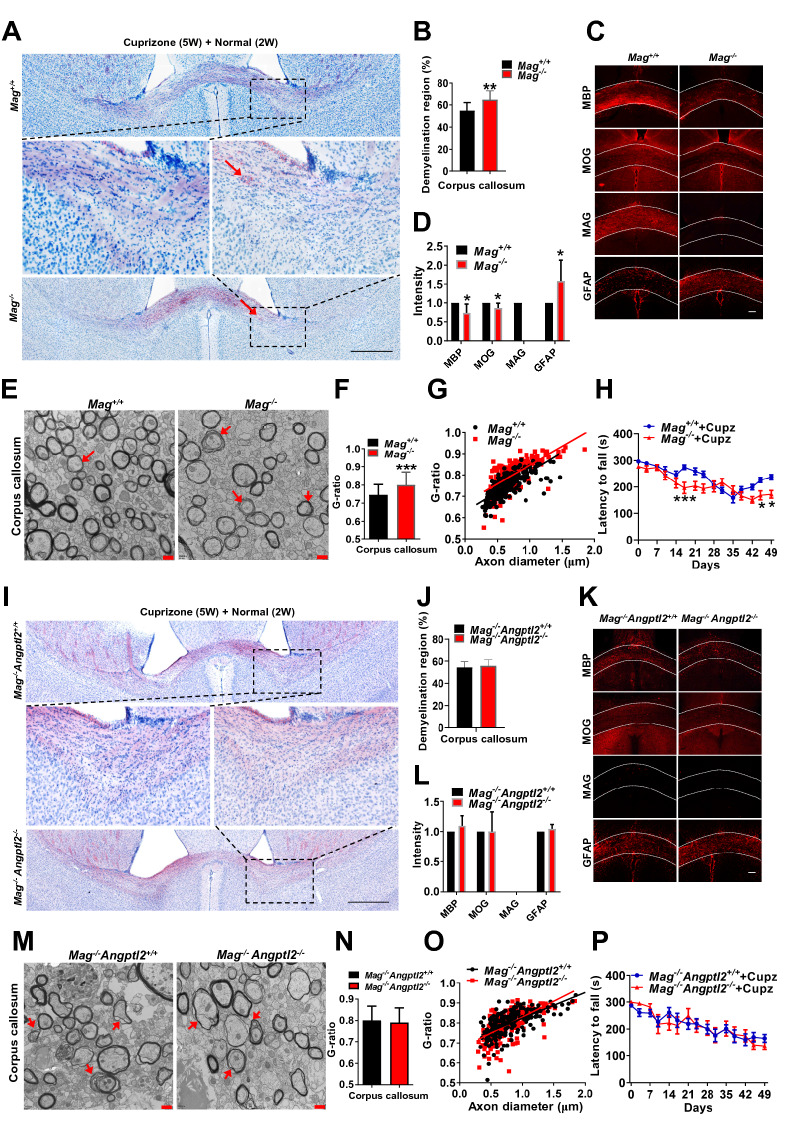
ANGPTL2 promotes remyelination via its receptor MAG. **A** Representative images of the corpus callosum region stained with oil red O in *Mag*^+*/*+^ and *Mag*^*−/−*^ mice after the withdrawal of cuprizone for two additional weeks. Myelin was stained in red color. Meanwhile, more nonspecific oil red O positive deposits (a phenomenon that oil red O deposits in damaged myelin region) were observed in *Angptl2*^*−/−*^ mice (arrows). **B** Quantification of the demyelinating areas in Panel A; six sections from each mouse were analyzed (n = 6). **C** Immunofluorescence images of MBP, MOG, MAG and GFAP staining in the corpus callosum region from *Mag*^+*/*+^ and *Mag *^*−/−*^ mice after the withdrawal of cuprizone for two additional weeks. **D** Quantification of the fluorescence intensity of MBP, MOG, MAG and GFAP in the corpus callosum in Panel C. Four sections from each mouse were analyzed (n = 6). **E** Representative electron microscopy images of the corpus callosum from *Mag*^+*/*+^ and *Mag*^*−/−*^ mice after the withdrawal of cuprizone for two additional weeks. Red arrow indicates uncompacted myelin lamella; **F** Quantification of the G ratios of the remyelinated axons of the corpus callosum in Panel **E**. Approximately 80 axons were counted per mouse (n = 3). **G** The scatter plot for the individual G-ratio values and axonal size distribution of the corpus callosum in Panel E. **H** Motor coordination for *Mag*^+*/*+^ and *Mag*^*−/−*^ mice after cuprizone treatment for five weeks and cuprizone (Cupz) withdrawal for two additional weeks in the rotarod test; 19–22 mice were used for each group. **I** Representative images of the corpus callosum region stained with oil red O in *Mag*^*−/−*^*Angptl2*^+*/*+^ and *Mag*^*−/−*^*Angptl2*^*−/−*^ mice after the withdrawal of cuprizone for two additional weeks. **J** Quantification of the demyelinating areas in Panel **I**; six sections from each mouse were analyzed (n = 3). **K** Immunofluorescence images of MBP, MOG, MAG and GFAP staining in the corpus callosum region from *Mag*^*−/−*^*Angptl2*^+*/*+^ and *Mag*^*−/−*^*Angptl2*^*−/−*^ mice after the withdrawal of cuprizone for two additional weeks. **L** Quantification of the fluorescence intensity of MBP, MOG, MAG and GFAP in the corpus callosum in Panel **K**; four sections from each mouse were analyzed (n = 3). **M** Representative electron microscopy images of the corpus callosum from *Mag*^*−/−*^*Angptl2*^+*/*+^ and *Mag*^*−/−*^*Angptl2*^*−/−*^ mice after the withdrawal of cuprizone for two additional weeks. Red arrow indicates uncompacted myelin lamella. **N** Quantification of the G ratios of the remyelinated axons of the corpus callosum in Panel **E**. Approximately 70 axons were counted per mouse (n = 3). **O** The scatter plot for the individual G-ratio values and axonal size distribution of the corpus callosum in Panel M. **P** Motor coordination in *Mag*^*−/−*^*Angptl2*^+*/*+^ and *Mag*^*−/−*^*Angptl2*^*−/−*^ mice after cuprizone (Cupz) treatment for 5 weeks and withdrawal of cuprizone for two additional weeks in the rotarod test; 8–13 mice were used for each group. (*p < 0.05, **p < 0. 01, ***p < 0. 001)

MAG deficiency induces a delay in oligodendrocyte differentiation and myelination formation at an early age [53], which is consistent with the phenotype of *Angptl2*-null mice under physiological conditions. We further investigated whether MAG was the functional receptor for ANGPTL2 during the cuprizone-induced demyelination and remyelination. There was no significant difference in demyelination levels between WT and *Mag*-null mice as measured by oil red O staining (Additional file 1: Fig. S5A–B) and immunohistochemistry (Additional file 1: Fig. S5C). Similar to the findings in *Angptl2*-null mice, *Mag*-null mice showed a slower recovery from acute demyelination than WT littermates with less myelin staining (while more nonspecific oil red O positive deposits) and lower expression levels of MBP and MOG (but higher GFAP level) (Fig. 5A–D). The G-ratio in axons under electron microscopy also revealed that MAG promoted remyelination 2 weeks after withdrawal of cuprizone chow (Fig. 5E–G), but no difference was observed during the progression of demyelination between these two groups after 5 weeks of cuprizone chow treatment (Additional file 1: Fig. S5D). *Mag*-null mice also had a much shorter latency to falling from the rotarod instrument than WT mice (168 s vs. 225 s at day 45, 171.8 vs. 236.6 s at day 49, Fig. 5H) after the withdrawal of cuprizone chow.

We generated *Mag*^*−/−*^*Angptl2*^+*/*+^ and *Mag*^*−/−*^*Angptl2*^*−/−*^ double mutant mice to test whether ANGPTL2 mediated enhanced myelination was dependent on the MAG receptor. Notably, *Mag*^*−/−*^*Angptl2*^+*/*+^ and *Mag*^*−/−*^*Angptl2*^*−/−*^ mice showed comparable remyelination capacities following cuprizone withdrawal as determined by oil red O staining (Fig. 5I–J), immunohistochemical staining (Fig. 5K–L) and electron microscopy (Fig. 5M–O). Similar demyelination levels in the corpus callosum were found between *Mag*^*−/−*^*Angptl2*^+*/*+^ and *Mag*^*−/−*^*Angptl2*^*−/−*^ mice 5 weeks after cuprizone chow treatment (Additional file 1: Fig. S5E–H). Notably, no significant differences in motor coordination or balance ability were observed between *Mag*^*−/−*^*Angptl2*^+*/*+^ and *Mag*^*−/−*^*Angptl2*^*−/−*^ mice in the cuprizone-induced mouse model (Fig. 5P), which suggested that ANGPTL2 fine-tuned oligodendrocyte differentiation by binding the MAG receptor to mediate downstream signaling under physiological and pathological situations.

### ANGPTL2-MAG induces Fyn-mediated signaling to enhance the differentiation of oligodendrocytes

**Fig. 6:**
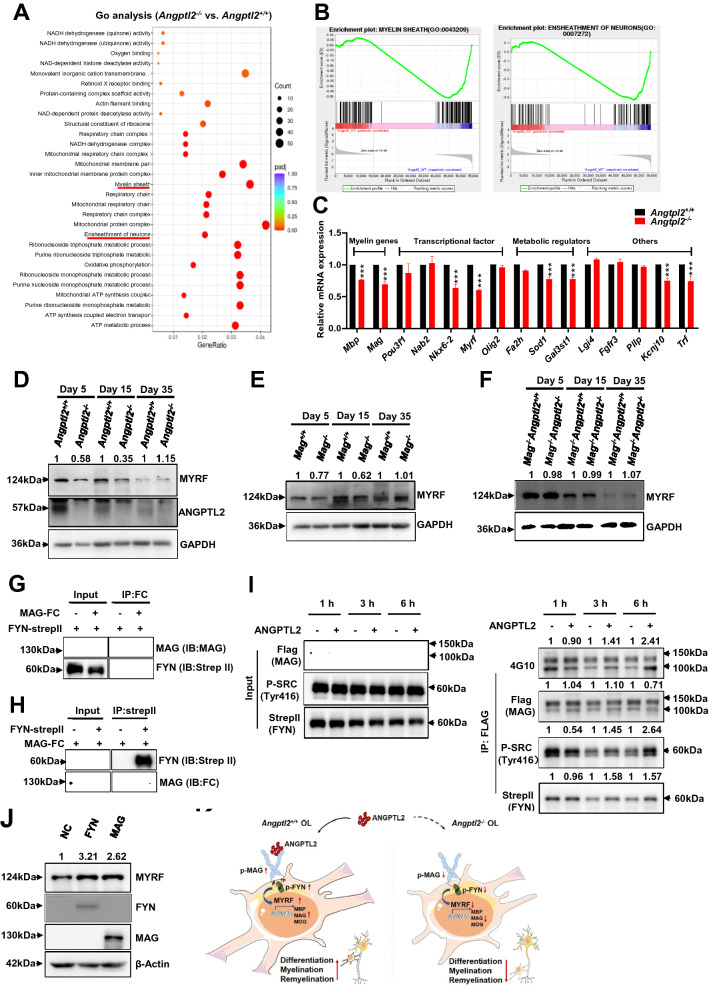
ANGPTL2-MAG induces Fyn-mediated signaling to enhance the differentiation of oligodendrocytes. **A** Gene Ontology (GO) analysis of the downregulated differentially expressed genes (DEGs) in the brains of *Angptl2*^+*/*+^ and *Angptl2*^*−/−*^ mice at day 15 as determined by RNA sequencing (n = 3). **B** Enrichment score plots from GSEA related to the GO signature for myelin sheath and ensheathment of neurons (n = 3). FDR, false discovery rate; NES, normalized enrichment score. **C** Relative mRNA levels of potential candidates related to myelination markers, transcription factors, metabolic regulators and other genes in the brain tissues of *Angptl2*^+*/*+^ and *Angptl2*^*−/−*^ mice at day 15 as measured by quantitative RT-PCR (n = 3). **D** Immunoblot analysis of MYRF and ANGPTL2 protein levels in the brain tissues of *Angptl2*^+*/*+^ and *Angptl2*^*−/−*^ mice at day 5, day 15 and day 35. Ratio of MYRF/β-actin was quantified and normalized against *Angptl2*^+*/*+^, respectively. One representative experiment is shown. **E–F** Immunoblot analysis of MYRF protein levels in the brain tissues of *Mag*^+*/*+^ and *Mag*^*−/−*^ mice (**E**) or *Mag*^*−/−*^*Angptl2*^+*/*+^ and *Mag*^*−/−*^*Angptl2*^*−/−*^ mice (**F**) at day 5, day 15 and day 35. Ratio of MYRF/β-actin was quantified and normalized against *Angptl2*^+*/*+^, respectively. One representative experiment is shown. **G**–**H** MAG directly interacted with FYN, as detected by forward (**G**) or reverse (**H**) co-immunoprecipitation assays. CMV-MAG (full-length)-FC and pLVX-FYN-strepII plasmids were used in this experiment. One representative experiment is shown. **I** RSC96 cells with ectopic expression of MAG (full-length)-FLAG and FYN-StrepII were treated with ANGPTL2 proteins, followed by co-immunoprecipitation analysis to evaluate the changes in tyrosine phosphorylation levels of MAG and FYN using 4G10 and p-SRC (Tyr416) antibodies, respectively. The levels of immunoprecipitated protein were quantified and normalized against the control group, respectively. One representative experiment is shown. **J** RSC96 cells overexpressing FYN-StrepII or MAG (full-length)-FC were subjected to immunoblot analysis to determine MYRF protein levels. Ratio of MYRF/β-actin was quantified and normalized against negative control (empty vector), respectively. One representative experiment is shown. **K** Western blot analysis of the protein levels of P-SRC (Tyr416), Fyn and MBP in HCN cells 72 h after induction with IGF1 (100 ng/ml), with/without ANGPTL2-Flag (2 μg/ml) and AZD0530 (2 μM) as indicated. Ratios of P-SRC (Tyr416)/β-actin, Fyn/β-actin, MYRF/β-actin and MBP/β-actin were quantified and normalized against the control treated with IGF1 alone, respectively. One representative experiment is shown. **L** Schematic diagram of the working model for the role of ANGPTL2-MAG in oligodendrocytes differentiation, myelination and differentiation. (***p < 0. 001)

To elucidate the ANGPTL2-MAG-mediated downstream signaling that controls oligodendrocyte differentiation and myelination, RNA-Seq analyses (GSE199393) were performed using the postnatal brains of WT and *Angptl2*-null mice at day 15. GO analysis showed that several pathways involved in oligodendrocyte differentiation or myelination (such as myelin sheath and ensheathment of neurons) (Fig. 6A–B) and related candidate genes (*Mbp, Mag, Pou3f1, Nab2, Nkx6-2, Myrf, Olig2, Fa2h, Sod1, Gal3st1, Lgi4, Fgfr3, Pllp, Kcnj10* and *Trf*) were notably decreased in *Angptl2*-null mice (Additional file 1: Fig. S6A). We further confirmed the mRNA expression levels of these candidate genes using quantitative RT-PCR and demonstrated that oligodendrocyte markers (*Mbp, Mag*), two key transcription factors (*Nkx6-2, Myrf*), metabolic regulators (*Sod1, Gal3st1*) and others (*Kcnj10*, *Trf*) were markedly downregulated (Fig. 6C). Because MYRF is critical for the differentiation and myelination of oligodendrocytes and enhance the expression of MAG, MBP and MOG [54, 55], we evaluated MYRF protein levels in the brains of *Angptl2*^+*/*+^, *Angptl2*^*−/−*^, *Mag*^+*/*+^, *Mag*^*−/−*^, *Angptl2*^+*/*+^ *Mag*^*−/−*^ and *Angptl2*^*−/−*^*Mag*^*−/−*^ mice. The results revealed that MYRF was downregulated in *Angptl2*^*−/−*^ and *Mag*^*−/−*^ mice at day 5 and day 15 compared to their littermates (Fig. 6D–E). However, there was no difference in MYRF protein levels between *Angptl2*^+*/*+^ *Mag*^*−/−*^ and *Angptl2*^*−/−*^*Mag*^*−/−*^ mice (Fig. 6F), which suggested that MYRF was the downstream candidate target of ANGPLT2-MAG signaling.

Fyn tyrosine kinase is a downstream signal of MAG and plays a critical role in oligodendrocyte differentiation and myelination [56]. However*,* the detailed mechanisms are not known. We demonstrated that MAG directly interacted with Fyn using a co-immunoprecipitation assay (Fig. 6G–H). MAG was overexpressed in an oligodendrocyte cell line, RSC96, and stimulated with ANGTPL2 to analyze the phosphorylation levels of MAG and Fyn, which are required for the initiation of downstream signaling cascades. Notably, MAG tyrosine phosphorylation levels increased markedly over time, as detected by 4G10 antibodies (Fig. 6I). The immunoprecipitated total and phosphorylation Fyn levels (p-Fyn was determined by the p-SRC416 antibody [57] due to lacking of its specific antibody) were also enhanced in the presence of ANGPTL2 (Fig. 6I). The overexpression of MAG and FYN upregulated the protein levels of MYRF (Fig. 6J). Using in vitro oligodendrocyte induction system with HCN cells, we found purified ANGTPTL2 protein significantly increased the Fyn phosphorylation level as detected by p-SRC (Tyr416) and the expression level of the downstream targets of MYRF and MBP, which can be abolished by adding the FYN inhibitor, AZD0530 (Fig. 6K, Additional file 1: Fig. S6E–F). Moreover, we also found that there were more mature oligodendrocytes with more web-like branches in morphology in the group supplemented with ANGPTL2 compared to the control group, which could be blocked by adding AZD0530 (Additional file 1: Fig.S6C–D). These results further suggested that ANGPTL2 promoted oligodendrocyte maturation mainly through Fyn-mediated signaling pathways. The phosphorylation levels of Fyn were markedly decreased in the brains of *Angptl2*-null mice on day 5 and day 15, but not day 35 (Additional file 1: Fig. S6B). The protein levels of MBP and MAG were also reduced in *Angptl2*-null mice at day 15, but not day 35 (Additional file 1: Fig. S6B), which suggested that ANGPTL2 deletion led to a transient delay in myelination. In summary, we demonstrated that ANGPTL2 bound MAG to enhance oligodendrocyte differentiation, myelination or remyelination capacity under physiological and pathological states, which was tightly regulated by the downstream Fyn/MYRF signaling (Fig. 6K). Our study provided a unique angle for understanding the oligodendrocyte differentiation and developing the potential strategy for the treatment of demyeliniation disorders in nerve systems.

## Discussion

ANGPTLs (ANGPTL1-8) are secretory proteins with similar structures to angiopoietin proteins and have multiple functions in angiogenesis, tissue repair [26, 29, 58], lipid metabolism, inflammation [28, 59], cardiovascular diseases [60, 61] and cancer development [34, 62]. Unlike the angiopoietin proteins, ANGPTL2 do not bind Tie1 or Tie2 [26], which suggests that ANGPTL2 play different roles by binding unknown receptors. For example, Oike et al. demonstrated that ANGPTL2 bound integrin α5β1 on adipocytes, endothelial cells and cancer cells to promote cell motility via Rac-mediated signaling [29]. CD146 was identified as a novel ANGPLT2 receptor manipulating lipid metabolism and energy expenditure [63]. We showed that LILRB2 was the receptor for ANGPTL2 to sustain the stemness of HSCs and leukemia stem cells [36]. We further revealed that ANGPTL2 bound to MAG on oligodendrocytes to enhance their differentiation via Fyn/MYRF mediated signals.

The current study identified MAG as another important receptor for ANGPTL2, and MAG was highly expressed on oligodendrocytes with a high binding affinity (KD ≈ 10 nM), which was comparable to the binding affinity between ANGPTL2 and LILRB2 (KD = 5.5 nM). Notably, LILRB2 was rarely expressed on oligodendrocytes (https://www.proteinatlas), which indicated that ANGPTL2 exerted its effects primarily via MAG-mediated pathways. These findings provide deep insight into the potential roles of ANGPTL2 in the regulation of oligodendrocyte differentiation and demyelinating disorders. However, the cell types in the CNS that primarily provide ANGPTL2 for oligodendrocyte differentiation are largely unknown. Although ANGPTL2 is primarily secreted by endothelial cells [64], macrophages [65] or adipocytes [29], public databases also indicate that oligodendrocyte precursor cells and oligodendrocytes have the highest expression levels of ANGTPL2 in the human brain (https://www.proteinatlas). However, the expression profile maybe different between human and mouse brains. Further efforts are required for the identification of the potential cell types (including oligodendrocyte precursor cells and oligodendrocytes) that contribute to ANGPTL2 levels in the brain for oligodendrocyte differentiation and myelination and the difference of ANGPTL2 expression profiles between human and mouse brains must be elucidated.

We also found that relatively high ANGPTL2 protein level was still expressed with oligodendrocyte differentiation from HCN cells in vitro, which suggested that the cell-autonomous effect might play a role in oligodendrocyte differentiation. We currently are testing its potential autocrine effect by knocking down Angptl2 in HCN cells during in vitro differentiation. Meanwhile, other cell types in the brain may be alternative sources for ANGPTL2 to trigger MAG-mediated signaling and promote oligodendrocyte differentiation in a paracrine manner. Interestingly, it seemed that MAG was only expressed on the differentiated oligodendrocytes, but not oligodendrocyte precursor cells (Additional file 1: Fig. S3G–H), and ANGPTL2 had no effect on the proliferation and differentiation of oligodendrocyte precursor cells (Additional file 1: Fig. S3F–G). Meanwhile, ANGPTL2 was also highly expressed in oligodendrocyte precursor cells (Additional file 1: Fig. S3D–E), suggested that oligodendrocyte precursor cells might secreted ANGPTL2 to support oligodendrocyte maturation in a paracrine manner. However, due to the lack of the suitable ANGPTL2 antibody for immunohistochemistry, more efforts are required to delineate the expression patterns of ANGPTL2 in the mouse brain. Moreover, we found that both of ANGPTL2 and MYRF level were gradually declined with ages (Fig. 6D), which might suggest that MYRF level was controlled by ANGPTL2/MAG signaling in an ANGPTL2 dose dependent manner. We expect that ANGPTL2 may mainly promote oligodendrocyte differentiation at the early stage or the process of remyelination, when large amounts of mature oligodendrocytes are required for rapid myelination. However, it is very difficult to perform rescue experiment in neonatal mice because ANGPTL2 may be not effectively transferred or reached enough amount in specific microenviroment to support oligodendrocyte maturation after ICV injection. Similarly, IP injection may also result in the failure in delivering ANGPTL2 into specific microenviroment for oligodendrocyte maturation due to the existence of blood–brain barrier. Other ways to increase ANGPTL2 level in brains are required to further delineate the function of ANGPTL2 in oligodendrocyte maturation. The transient phenotype in developmental myelination in knockout animals indicates that ANGPTL2 is not fully necessary for full differentiation, and neither the maintenance of normal myelin in the adult animals. It will be also important to elucidate how ANGPTL2 affects oligodendrocyte differentiation at different stages and whether other ANGPTLs are required for differentiation.

MAG acts as a glue or spacer for glia and axons [66, 67] and plays an important role in the maintenance of glia-axon communication. However, the roles and detailed mechanisms of MAG in myelin formation are controversial. Li et al. reported that MAG was not critical for myelin formation, but it was required for the maintenance of the cytoplasmic collar and periaxonal space of myelinated axons in adult mice [44]. However, Pernet et al. found a delayed oligodendrocyte differentiation and abnormal myelin structure in central nervous system of *Mag*-null mice within the first month after birth [53]. We showed that ANGPTL2-MAG signaling enhanced oligodendrocyte differentiation and myelination at the early stage and remyelination progression, under certain pathological stresses. These results strengthen the hypothesis that MAG is critical for the homeostasis of the oligodendrocyte pool and its myelination function.

MAG has been reported to be a ligand for the Nogo receptor (NgR) and PirB, which mainly involves in the inhibition of axon elongation [39–41, 68]. For example, MAG and Nogo66 can compete for binding to NgR to inhibit neurite outgrowth [39, 68], PirB is another receptor for MAG, Nogo66 and OMgp to serve as the inhibitor in axonal regeneration [40]. MAG serves as a receptor for nerve cell surface gangliosides GD1a and GT1b to mediate nerve regeneration inhibition [42]. Interestingly, MAG also can form the complex with β1-integrin to mediate axonal growth cone repulsive response of hippocampal neurons independent of NgR through FAK activation [41]. However, except for its inhibitory effect on axon growth, whether MAG can serve as a receptor to mediate the downstream signaling to enhance the oligodendrocyte differentiation is not clear. Herein, we demonstrated that ANGPTL2 served as the potent ligand for MAG and enhanced oligodendrocyte differentiation by increasing the phosphorylation of MAG to recruit and activate the non-receptor tyrosine kinase Fyn to enhance the expression of the downstream key transcription factor MYRF and its targets of some myelin-related markers, such as MBP, MAG and MOG [54, 55]. These results provide a unique angle to understand the multifaceted functions of MAG via individual interactions with certain surface molecules. However, how MAG activates Fyn kinase and the downstream target MYRF, which further connect to the differentiation and myelination of oligodendrocytes, is largely unknown.

## Materials and methods

### Animals

The *Angptl2* knockout mice (*Angptl2*^*fl/fl*^) with insertion of loxp in flanks of exon 2 in C57BL/6 background were generated by Nanjing Mouse Model, Lt Corporation. The male *Angptl2*^*fl/fl*^; *stra8-cre* mice (*Angptl2* is specifically deleted in early-stage spermatogonia, spermatocytes and sperm) were crossed with wild-type (WT) female mice to generate *Angptl2* knockout mice (*Angptl2*^*−/−*^). The genetic deletion of *Angptl2* was further confirmed by PCR. The *Mag* knockout mice (*Mag*^*−/−*^) in C57BL/6 background were purchased from Mutant Mouse Regional Resource Centers (MMRRC). *Mag*^*−/−*^*Angptl2*^*−/−*^ double knockout mice and their littermates *Mag*^*−/−*^*Angptl2*^+*/*+^ mice were also bred for the related experiments. The Guideline for Animal Care at Shanghai Jiao Tong University School of Medicine approved all the animal experimental procedures.

### Murine cuprizone-induced demyelination model and rotarod test

The 8–10 week old mice were fed with a standard rodent chow with 0.25% (w/w) cuprizone (bis (cycloheanone) oxaldihydrazone; Sigma) for 5 weeks to induce the demyelination in mouse brains, followed by the switch to the normal chow for 2 additional weeks to allow the recovery from demyelination. The cuprizone-induced demyelinated or remyelinated mice were anesthetized, perfused with PBS, followed by fixation with 4% PFA. The whole mouse brains were removed, post-fixed, sectioned at 30 μm using a vibratome, and subjected for the oil-red O staining, immunohistochemical analysis and electron microcopy analysis. In consideration of the cuprizone induced lesions are in the variability in lesion size and location, the corresponding section every ten serial coronal sections in the corpus callosum of mice was selected and 6–8 sections in one brain were stained for analysis. For the motor coordination assessment, the 8–10 week old mice first received a training of running on a rotating rod at an accelerating speed from 4 to 40 rotations per min for 300 s for 1 week (Harvard apparatus, UK). The mice that could still stay on the rotating cylinder at a speed of 4 rotations/min for 300 s were used for the following motor coordination analysis. The latency to fall off a rotating rod at a speed of 4 rotations/min and the body weight of each mouse were measured every 3 days during the 5 weeks’ cuprizone chow feeding and following 2 weeks’ normal chow feeding.

### Oil-red O staining and demyelination area scoring

Oil-red O Assay Kit (BASO Life Technologies, BA4081) was used for the evaluation of myelination status according to the manufacturer’s instructions. Briefly, coronal brain slices were rehydrated with distilled water for 5 min, treated with 60% isopropanol for 2 min and rinsed in oil red O for 10 min. Excess stain was removed by washing the slides with 60% isopropanol followed by washing with distilled water. Slices were counterstained with hematoxylin to visualize nuclei. Images were taken using an Olympus IX71 inverted fluorescence microscope and quantitative analysis was performed using Olympus cellsens. Region of interest at corpus callosum was drawn using the “irregular AOI” tool and red areas were counted within the lesion areas using the “count and measure objects” tool. Percentage of the demyelination area was calculated by the ratio of the unstained area and total corpus callosum area.

### Immunofluorescence staining and immunohistochemistry

For immunofluorescence staining, coronal brain slices were blocked with permeable buffer (0.3% Triton X-100 in PBS) containing 10% donkey serum for an hour at room temperature and incubated with primary antibodies in permeable buffer containing 2% donkey serum overnight at 4 °C. The slices were then washed three times with PBS-T (0.1% Tween 20 in PBS) for 10 min each time and incubated with Alexa Fluor secondary antibodies (Thermo Fisher) in the PBS for 2 h at room temperature. Nuclei were counterstained with DAPI (Invitrogen). For primary antibodies, rabbit anti-MBP (CST, Cat#78896), mouse anti-MAG (Abcam, ab89780), mouse anti-MOG polyclonal antibody (Beyotime, AF7488 and Santa Cruz Biotech, SC-166172), rabbit anti-GFAP and (CST, Cat#12389). Images were taken using an Olympus IX71 inverted fluorescence microscope, and quantitative image analysis was performed using Image J.

In some cases, an adult hippocampus-derived neural progenitor cell line, HCN cells, was used for the analysis of ANGPTL2-mediated oligodendrocyte differentiation. HCN cells were plated on ornithine-pretreated glass, fixed with 4% PFA for 10 min at room temperature, blocked with TBS containing 0.1% Triton and 2% BSA, and incubated with primary antibodies, including rabbit anti-MBP (CST, Cat#78896), mouse anti-MOG (Beyotime, AF7488 and Santa Cruz Biotech, SC-166172), mouse anti-MAG (Abcam, ab89780), mouse anti-PDGFRα (R&D, AF1062) and mouse anti-Ki-67-FITC (BD, Cat#612472) at 4 °C overnight. After washing with PBS for three times, HCN cells were incubated with the appropriated secondary antibody conjugated with Alexa Fluor 555 (Thermo Fisher) for one hour at room temperature. Nuclei were counterstained with DAPI (Invitrogen).

### Electron microscopy analysis

Mice were perfused with the PBS buffer containing 2.5% PFA and 2.5% glutaraldehyde and mouse brains were isolated for the subsequent electron microscopy analysis. Corpus callosum were washed, fixed in 1% osmium tetroxide, dehydrated in acetone and embedded in EPON. A 70-nm thinness sagittal section was cut with a diamond knife, mounted on copper slot grids precoated with Formvar and stained with uranyl acetate and lead citrate for the examination of myelination with Hitachi H-7650 transmission electron microscope. G-ratios were determined using Image J software. Approximately 120–200 axons in each mouse (three mice per group) were analyzed and the total numbers of axons counted were about 360–600 in each group. The significant difference was analyzed by Unpaired Student’s t test (two-tailed) as shown in Fig. 3F, H, and 200 ~ 500 remyelinated axons were calculated for each group in Fig. 4F, 5F, N.

### Cell culture

293T cells were cultured in Dulbecco’s modified Eagle’s medium (DMEM) supplemented with 10% FBS. An adult hippocampus-derived neural progenitor cell line, HCN cells [46, 69], were cultured in DMEM-F12 (Gibco) plus N2 supplement (Gibco, 17502-048), L-Glutamine (Gibco, A2916801), 20 ng/ml bFGF (Peprotech, AF-100-18B) and penicillin–streptomycin (P/S). To induce the differentiation of HCN cells to mature oligodendrocytes, HCN cells were carefully digested with 0.05% trypsin, neutralized with defined trypsin inhibitor (Gibco, R-007-100), washed with PBS to get rid of all the remaining growth factor of bFGF, followed by culturing in the medium of DMEM-F12 plus 100 ng/ml IGF1 (Peprotech, 100-11), N2 supplement (Gibco, 17502-048), L-Glutamine (Gibco, A2916801) and P/S for additional 3 days. All the plates for the cell growth and differentiation of HCN cells were pretreated a with poly-ornithine (Sigma, P3655) and murine laminin (Invitrogen, 23017–015).

### Flow cytometric analysis for the ANGPTL2 binding activities to MAG

CMV-ANGPTL2-Flag plasmid or other plasmids of ANGPTL members [36] were transfected into 293T cells and the conditioned medium was collected 48 h after transfection for the binding assay with MAG or the purification of ANGPTL2 using M2 resin (Sigma, A2220). Human or murine MAG protein fused with GFP were cloned in the CMV-N1-GFP plasmid and transfected into 293T cells. The 293T cells were collected 48 h after transfection and incubated with ANGPTL2-Flag condition medium or control medium at 4 °C for 1–2 h, followed by the incubation with the secondary antibody of anti-Flag-allophycocyanin (APC) (Biolegend, Cat# 637308) and propidium iodide (PI). The potential binding activities of ANGPTL2 to MAG were determined by flow cytometric analysis.

### Selecting surface molecules for ANGPTL2

The selecting surface molecules were mainly based on a flow cytometric assay. The membrane protein library contained individual expression plasmid without reporting tags, such as GFP or mCherry. To indicate the expression level of transfected surface molecules, the fluorescence indictor of XZ201-GFP plasmid was co-transfected with individual membrane plasmid. Individual membrane plasmid (0.25 µg) and XZ201-GFP plasmid were mixed at ratio of 1:1 and transiently transfected into 293T cells. The transfected 293T cells were collected 48 h later and incubated with conditional medium containing ANGPTL2-FLAG protein at 4 °C for 1–2 h, followed by the incubation with the secondary antibody of anti-Flag-allophycocyanin (APC) and propidium iodide (PI). The potential binding activities of ANGPTL2 to membrane protein were determined by flow cytometric analysis. To prepare the ligand of ANGPTL2, the CMV-ANGPTL2-Flag plasmid was used for the transfection into 293T cells and the conditioned medium was collected 48 h later as described previously.

### Chimeric receptor reporter assay

Human or murine MAG chimeric receptor cells were established for the evaluation of the binding between ANGPTL2 and MAG according to the protocol previously described [43]. In brief, the ECDs of MAG and its mutants (IgG3 and IgG4) were fused to the intracellular domain PIRLb, which could further recruit adaptor DAP12 to transactivate NFAT-GFP expression upon binding to their potential ligands, such as ANGPTL2. In this assay, purified ANGPTL2 protein (0.02 mg/mL) was pre-coated on 96-well plate at 37 °C for 4–6 h, followed by culture with 4 × 10^4^ MAG reporter cells or control reporter cells in each well. The percentage of GFP^+^ reporter cells that represented the activation by ANGPTL2 binding to MAG were measured by flow cytometry 24 h after culture.

### Bio-layer interferometry or surface plasmon resonance

The binding affinity of human or murine MAG to ANGPTL2 was first determined by Octet RED96/Octet RED96e instrument (ForteBio). Condition medium containing human or murine MAG ECD fused with human Fc (MAG-hFC) was collected 48 h after transfection. The AHC biosensors were coated with MAG-hFc protein (condition medium containing human or mouse MAG-hFc protein was loaded on biosensors for 480 s/600 s), washed with kinetics buffer for 300 s/300 s before the determination of the association (300 s/90 s) and dissociation (800 s/90 s) constant upon binding to purified ANGPTL2 protein at indicated doses. Data were analyzed using ForteBio Data Analysis Software v9. Alternatively, Biacore T200 instrument (GE Healthcare) and CM5 sensor chips were used to determine the binding affinity of human or murine MAG-hFC to ANGPTL2 protein. Briefly, anti-hFc antibody (Sigma, Cat#12136) was pre-immobilized in parallel-flow channels of a CM5 sensor chip using the amine coupling kit (GE Healthcare). Human or murine MAG-hFc in the condition medium was injected into one of the channels and captured by the anti-hFc antibodies pre-coated on the CM5 sensor chip. To measure the binding affinity of human or murine MAG-hFc to ANGPTL2, indicated doses of purified ANGPTL2 protein were injected into the flow system. The binding affinity constants were analyzed with Biacore T200 evaluation software V3.

### RT-PCR

The total RNA of HCN cells and their differentiated cells, or *Angptl2*^+*/*+^ and *Angptl2*^*−/−*^ mouse brain tissues were extracted and used for real-time RT-PCR. The reactions were performed as previously described [70]. Briefly, 10 μL reactions with 2 × ABI SYBR^®^ Green PCR master mix, primers and cDNA were used for the evaluation of indicated gene expression levels. The experiments were conducted in triplicate with Applied Biosystems 7900HT. The mRNA level was normalized to the level of β-actin RNA transcripts. The primer sequences for related genes were showed in Additional files 2. The unedited agarose gel figures were showed in Additional file 3.

### Co-immunoprecipitation

Plasmids encoding human or murine MAG-ECD-Fc, Tie2-ECD-Fc, ANGPTL2-strep II and control vector were transiently co-transfected into 293T cells. The supernatant were collected 48 h after transfection and incubated with Protein A/G beads (Santa Cruz, sc2003) at 4 °C for 8 h, followed by washing with pre-chilled PBS with 0.1% NP-40 for 5 times. In the co-immunoprecipitation experiment for FYN and MAG, 293 T cells were transiently co-transfected with MAG (full-length)-FC, FYN-strepII and control vectors, lysed and incubated with Protein A/G beads (Santa Cruz, sc2003) or anti-strepII (GenScript, Cat#A00626-40) at 4 °C overnight, followed by washing with pre-chilled PBS with 0.1% NP-40 for 8 times. The immunoprecipitated proteins were examined by using the indicated antibodies for anti-strepII (GenScript, Cat#A00626-40) or anti-Fc (Sigma, Cat#12136) by western blot. The unedited agarose gel figures were showed in Additional file3

### Western blot

Brain tissues isolated from *Angptl2*^*−/−*^, *Mag*^*−/−*^, *Mag*^*−/−*^*Angptl2*^+*/*+^, *Mag*^*−/−*^*Angptl2*^*−/−*^ and their controls were homogenized with RIPA lysis buffer (Beyotime, Cat#P0013C; supplemented with 1 mM PMSF, 2 mM sodium orthovanadate and protease inhibitor) at 4 °C for 30 min, and ultrasonicated for western blot analysis. Samples were separated by SDS-PAGE, transferred to nitrocellulose membranes and immunoblotted with primary antibodies as following: anti-MBP (CST, Cat#78896), anti-MAG (Abcam, ab89780), anti-MOG polyclonal antibody (Santa Cruz Biotech, SC-166172), anti-Fyn (Abcam, ab184276), anti-Phospho-Src family (Tyr416) (CST, Cat #2101), anti-MYRF (Abclonal, A16355), anti-ANGPTL2 (R&D, AF1444) and anti-β-Actin-pAb-HRP-DirecT (MBL, PM053-7). The unedited western blot gel figures were showed in Additional file 3.

### Statistical analysis

GraphPad and SPSS software programs, version 19.0, were used for statistical analysis. The results are presented as mean ± SD. The data were analyzed by Student’s t test (two-tailed), one-way ANOVA with Tukey’s multiple comparison test, or two-way ANOVA with Sidak’s multiple comparison test according to the experimental design. All experiments were performed independently more than 3 times. For all experiments, *p ≤ 0.05 was considered a significant difference (*p < 0.05; **p < 0.01; ***p < 0.001).

## Supplementary Information

**Additional file 1: Figure S1.** Related to Fig. 1. Human MAG is a new receptor for ANGPTL2. **Figure S2.** related to Fig. 2. Murine homologue of human MAG binds to ANGPTL2. **Figure S3.** Related to Fig. 3. ANGPTL2 promotes oligodendrocyte differentiation. **Figure S4.** Related to Fig. 4. ANGPTL2 promotes oligodendrocyte differentiation and remyelination under pathological conditions. **Figure S5.** Related to Fig. 5. ANGPTL2 promotes remyelination via its receptor MAG. **Figure S6.** Related to Fig. 6. ANGPTL2-MAG induces Fyn-mediated signalling to enhance the differentiation of oligodendrocytes.

**Additional file 2: Table S1.** Primer sequences for q-RT-PCR and genotyping.

**Additional file 3: **Unedited images from gels and western blots.

## Data Availability

For original data, please contact yapingzhang@shsmu.edu.cn. RNA sequencing data are available at GEO under accession number GSE199393.
