# Correction: ANGPTL2 binds MAG to efficiently enhance oligodendrocyte differentiation

**DOI:** 10.1186/s13578-023-01013-7

**Published:** 2023-03-30

**Authors:** Lu Chen, Zhuo Yu, Li Xie, Xiaoxiao He, Xingmei Mu, Chiqi Chen, Wenqian Yang, Xiaoping Tong, Junling Liu, Zhengliang Gao, Suya Sun, NanJie Xu, Zhigang Lu, Junke Zheng, Yaping Zhang

**Affiliations:** 1 Hongqiao International Institute of Medicine, Shanghai Tongren Hospital/Faculty of Basic Medicine, Key Laboratory of Cell Differentiation and Apoptosis of Chinese Ministry of Education, Shanghai Jiao Tong University School of Medicine, 280 South Chongqing Road, Shanghai, 200025 China; 2 Center for Brain Science, Shanghai Children’s Medical Center, Department of Anatomy and Physiology, Shanghai Jiao Tong University School of Medicine, Shanghai, China; 3 Department of Biochemistry and Molecular Cell Biology, Shanghai Key Laboratory of Tumor Microenvironment and Inflammation, Shanghai Jiao Tong University School of Medicine, Shanghai, China; 4 Yangzhi Rehabilitation Hospital (Shanghai Sunshine Rehabilitation Center), Tongji Univeirsity School of Medicine, Shanghai, China; 5 Department of Neurology and Institute of Neurology, Ruijin Hospital, Shanghai Jiao Tong University School of Medicine, Shanghai, China; 6 Collaborative Innovation Center for Brain Science, Department of Anatomy and Physiology, Key Laboratory of Cell Differentiation and Apoptosis of the Chinese Ministry of Education, Shanghai Key Laboratory of Reproductive Medicine, Shanghai Jiao Tong University School of Medicine, Shanghai, China; 7 The Fifth People’s Hospital of Shanghai, the Shanghai Key Laboratory of Medical Epigenetics, The International Co-Laboratory of Medical Epigenetics and Metabolism, Ministry of Science and Technology, Institutes of Biomedical Sciences, Shanghai Institute of Infectious Diseases and Biosecurity, Shanghai Medical College, Fudan University, Shanghai, China

**Correction: Cell & Bioscience (2023) 13:42** **https://doi.org/10.1186/s13578-023-00970-3**

In this original article [1], the wrong figure appeared as Fig. 6 and Fig. 6K is missing. The correct Fig. 6 should have appeared as shown.

**Fig. 6:**
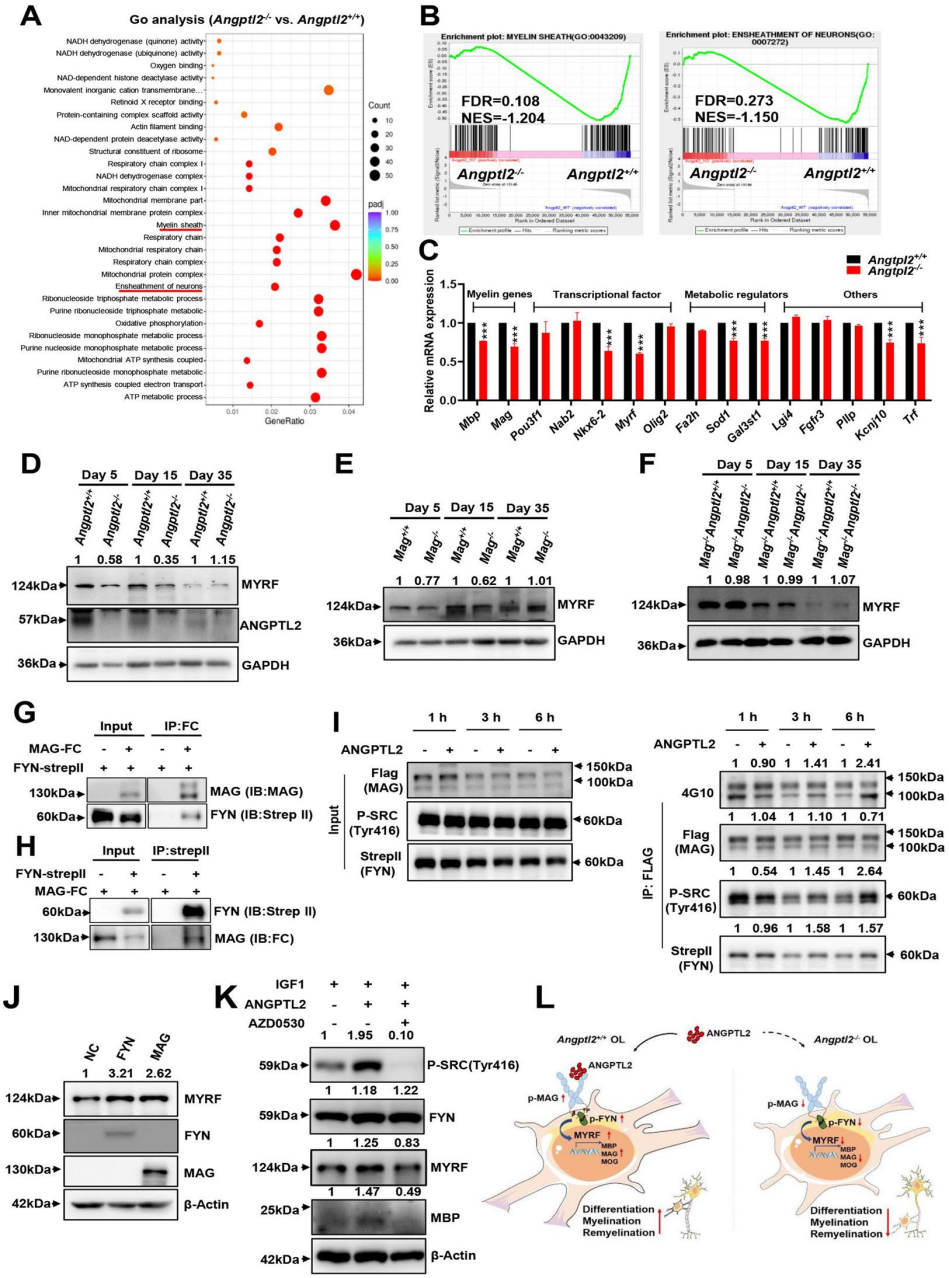
ANGPTL2-MAG induces Fyn-mediated signaling to enhance the differentiation of oligodendrocytes. **A** Gene Ontology (GO) analysis of the downregulated differentially expressed genes (DEGs) in the brains of *Angptl2*^+*/*+^ and *Angptl2*^*−/−*^ mice at day 15 as determined by RNA sequencing (n = 3). **B** Enrichment score plots from GSEA related to the GO signature for myelin sheath and ensheathment of neurons (n = 3). FDR, false discovery rate; NES, normalized enrichment score. **C** Relative mRNA levels of potential candidates related to myelination markers, transcription factors, metabolic regulators and other genes in the brain tissues of *Angptl2*^+*/*+^ and *Angptl2*^*−/−*^ mice at day 15 as measured by quantitative RT-PCR (n = 3). **D** Immunoblot analysis of MYRF and ANGPTL2 protein levels in the brain tissues of *Angptl2*^+*/*+^ and *Angptl2*^*−/−*^ mice at day 5, day 15 and day 35. Ratio of MYRF/β-actin was quantified and normalized against *Angptl2*^+*/*+^, respectively. One representative experiment is shown. **E–F** Immunoblot analysis of MYRF protein levels in the brain tissues of *Mag*^+*/*+^ and *Mag*^*−/−*^ mice (**E**) or *Mag*^*−/−*^*Angptl2*^+*/*+^ and *Mag*^*−/−*^*Angptl2*^*−/−*^ mice (**F**) at day 5, day 15 and day 35. Ratio of MYRF/β-actin was quantified and normalized against *Angptl2*^+*/*+^, respectively. One representative experiment is shown. **G**–**H** MAG directly interacted with FYN, as detected by forward (**G**) or reverse (**H**) co-immunoprecipitation assays. CMV-MAG (full-length)-FC and pLVX-FYN-strepII plasmids were used in this experiment. One representative experiment is shown. **I** RSC96 cells with ectopic expression of MAG (full-length)-FLAG and FYN-StrepII were treated with ANGPTL2 proteins, followed by co-immunoprecipitation analysis to evaluate the changes in tyrosine phosphorylation levels of MAG and FYN using 4G10 and p-SRC (Tyr416) antibodies, respectively. The levels of immunoprecipitated protein were quantified and normalized against the control group, respectively. One representative experiment is shown. **J** RSC96 cells overexpressing FYN-StrepII or MAG (full-length)-FC were subjected to immunoblot analysis to determine MYRF protein levels. Ratio of MYRF/β-actin was quantified and normalized against negative control (empty vector), respectively. One representative experiment is shown. **K** Western blot analysis of the protein levels of P-SRC (Tyr416), Fyn and MBP in HCN cells 72 h after induction with IGF1 (100 ng/ml), with/without ANGPTL2-Flag (2 μg/ml) and AZD0530 (2 μM) as indicated. Ratios of P-SRC (Tyr416)/β-actin, Fyn/β-actin, MYRF/β-actin and MBP/β-actin were quantified and normalized against the control treated with IGF1 alone, respectively. One representative experiment is shown. **L** Schematic diagram of the working model for the role of ANGPTL2-MAG in oligodendrocytes differentiation, myelination and differentiation (***p < 0. 001)

The original article has been corrected.
