# Supplementary material for: ANGPTL2 binds MAG to efficiently enhance oligodendrocyte differentiation
Source: Cell Biosci. 2023 Feb 28;13:42. doi: 10.1186/s13578-023-00970-3 (PMC9976406; doi:10.1186/s13578-023-00970-3)
Supplement: Supplementary file 1 — Additional file 1: Figure S1. Related to Fig. 1. Human MAG is a new receptor for ANGPTL2. Figure S2. related to Fig. 2. Murine homologue of human MAG binds to ANGPTL2. Figure S3. Related to Fig. 3. ANGPTL2 promotes oligodendrocyte differentiation. Figure S4. Related to Fig. 4. ANGPTL2 promotes oligodendrocyte differentiation and remyelination under pathological conditions. Figure S5. Related to Fig. 5. ANGPTL2 promotes remyelination via its receptor MAG. Figure S6. Related to Fig. 6. ANGPTL2-MAG induces Fyn-mediated signalling to enhance the differentiation of oligodendrocytes. [file 13578_2023_970_MOESM1_ESM.pdf]

Figure S1

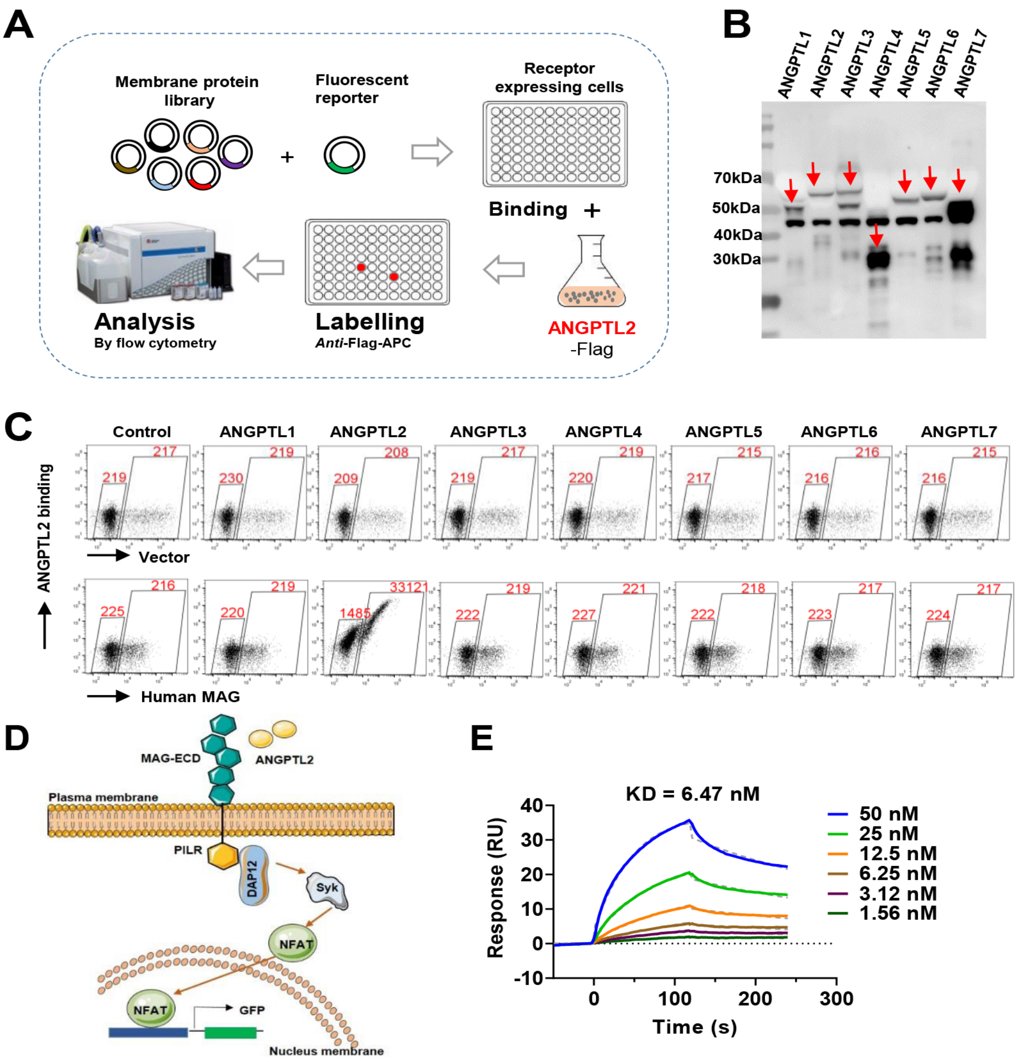

A
Membrane protein library
Fluorescent reporter
Receptor expressing cells
Binding +
ANGPTL2
-Flag
Labelling
Anti-Flag-APC
Analysis
By flow cytometry
B
ANGPTL1
ANGPTL2
ANGPTL3
ANGPTL4
ANGPTL5
ANGPTL6
ANGPTL7
70kDa
50kDa
40kDa
30kDa
C
Control
ANGPTL1
ANGPTL2
ANGPTL3
ANGPTL4
ANGPTL5
ANGPTL6
ANGPTL7
ANGPTL2 binding
Vector
Human MAG
D
MAG-ECD
ANGPTL2
Plasma membrane
PILR
DAP12
Syk
NFAT
NFAT
GFP
Nucleus membrane
E
KD = 6.47 nM
Response (RU)
Time (s)
50 nM
25 nM
12.5 nM
6.25 nM
3.12 nM
1.56 nM

Figure S1. related to Figure 1. Human MAG is a new receptor for ANGPTL2. (A) Schematic diagram for the strategy in screening of potential receptors for ANGPTL2 with a membrane protein library. (B) Protein levels of ANGTPTL members (ANGPTL1-7) in conditioned medium were examined using Western blotting and an anti-Flag antibody. (C) Flow cytometric analysis of the binding of ANGPTL1-7 to N1-MAG-EGFP (human MAG) or N1-EGFP (vector) in transiently transfected 293T cells. MFIs are indicated in red. (D) Schematic diagram of chimeric receptor cells with human MAG ECD. (E) Binding kinetics of ANGPTL2 to human MAG-ECD were measured using surface plasmon resonance (SPR).

Figure S2

A

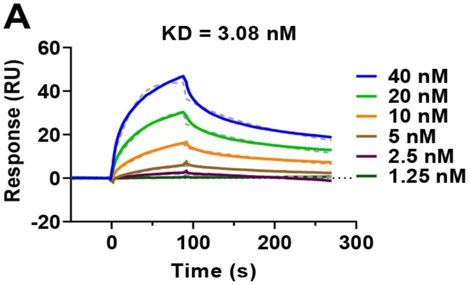
KD = 3.08 nM
Response (RU)
60
40
20
0
-20
0
100
200
300
Time (s)
40 nM
20 nM
10 nM
5 nM
2.5 nM
1.25 nM

Figure S2. related to Figure 2. Murine homologue of human MAG binds to ANGPTL2. (A) Binding kinetics of ANGPTL2 to murine MAG-ECD were measured using surface plasmon resonance (SPR).

Figure S3

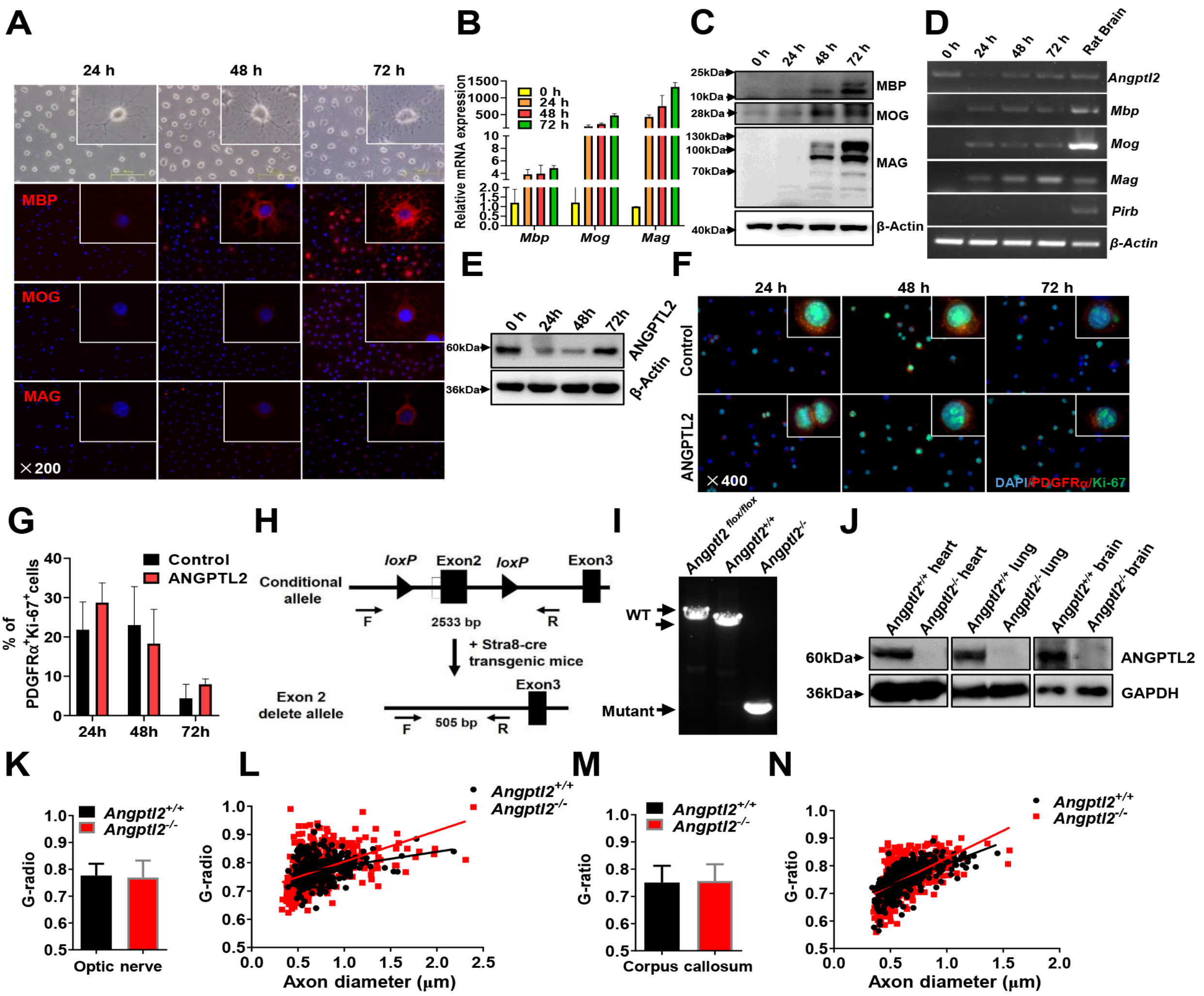

A
24 h
48 h
72 h
MBP
MOG
MAG
×200
B
Relative mRNA expression
0 h
24 h
48 h
72 h
Mbp
Mog
Mag
C
0 h
24 h
48 h
72 h
25kDa
10kDa
28kDa
130kDa
100kDa
70kDa
40kDa
MBP
MOG
MAG
β-Actin
D
0 h
24 h
48 h
72 h
Rat Brain
Angptl2
Mbp
Mog
Mag
Pirb
β-Actin
E
0 h
24h
48h
72h
60kDa
36kDa
ANGPTL2
β-Actin
F
24 h
48 h
72 h
Control
ANGPTL2
×400
DAPI/PDGFRα/Ki-67
G
% of PDGFRα+Ki-67+cells
Control
ANGPTL2
24h
48h
72h
H
Conditional allele
loxP
Exon2
loxP
Exon3
F
2533 bp
R
+ Stra8-cre transgenic mice
Exon 2 delete allele
Exon3
F
505 bp
R
I
Angptl2 flox/flox
Angptl2+/+
Angptl2-/-
WT
Mutant
J
Angptl2+/+ heart
Angptl2-/- heart
Angptl2+/+ lung
Angptl2-/- lung
Angptl2+/+ brain
Angptl2-/- brain
60kDa
36kDa
ANGPTL2
GAPDH
K
Angptl2+/+
Angptl2-/-
G-radio
Optic nerve
L
Angptl2+/+
Angptl2-/-
G-radio
Axon diameter (μm)
M
Angptl2+/+
Angptl2-/-
G-ratio
Corpus callosum
N
Angptl2+/+
Angptl2-/-
G-ratio
Axon diameter (μm)

Figure S3. related to Figure 3. ANGPTL2 promotes oligodendrocyte differentiation. (A) Cell morphology of HCN cells after IGF1 treatment *in vitro* at the indicated time points (1st row). Several oligodendrocyte markers (MBP, MOG and MAG) were used for immunofluorescence staining in HCN cells (2nd-4th rows). (B) Relative mRNA expression levels of *Mbp*, *Mog* and *Mag* in HCN cells after induction with IGF1 at the indicated time points using quantitative RT-PCR. (C) The protein levels of MBP, MOG and MAG in HCN cells after induction with IGF1 at the indicated time points were examined using Western blot. (D) The mRNA expression levels of *Angptl2, Mbp, Mog, Mag* and *Pirb* in HCN cells after induction with IGF1 at the indicated time points were examined using quantitative RT-PCR. Rat brain served as the positive control. (E) ANGPTL2 protein levels in HCN cells induced with IGF1 at the indicated time points were measured using Western blot. (F) Immunofluorescence staining for PDGFRα and Ki-67 in HCN cells after treatment with IGF1 *in vitro* with/without ANGPTL2 protein at the indicated time points. ANGPTL2 protein was added every 24 hours at the final concentration of 4 μg/mL. (G) PDGFRα^{+}Ki-67^{+} oligodendrocyte precursor cells in Panel F were calculated Quantitative data are shown; a total of 4 or 5 sections were counted. (H) Schematic diagram for the strategy for the construction of the *Angptl2* conditional knockout mouse lines (*Angptl2*$^{fl/fl}$), followed by crossbreeding with *Stra8-Cre* transgenic mice for the eventual generation of *Angptl2* knockout mice (*Angptl2*$^{-/-}$). (I) Representative electrophoretic images for the genotyping of *Angptl2*$^{fl/fl}$, *Angptl2*$^{+/+}$ and *Angptl2*$^{-/-}$ mice. (J) ANGPTL2 protein levels in different murine tissues, including the heart, lung and brain, from *Angptl2*$^{+/+}$ and *Angptl2*$^{-/-}$ mice as measured using Western blotting. (K-N) Quantification of the G ratios of the myelinated axons of the optic nerve fibers (K) or corpus callosum (M) of the brains from *Angptl2*$^{+/+}$ and *Angptl2*$^{-/-}$ mice at day 35. A total of 120~200 axons in each

mouse were counted (n=3). The scatter plots for the individual G-ratio values and axonal size distribution in the optic nerve fibers (L) or corpus callosum (N) in the brains from *Angptl2*$^{+/+}$ and *Angptl2*$^{-/-}$ mice at day 35 are shown.

Figure S4

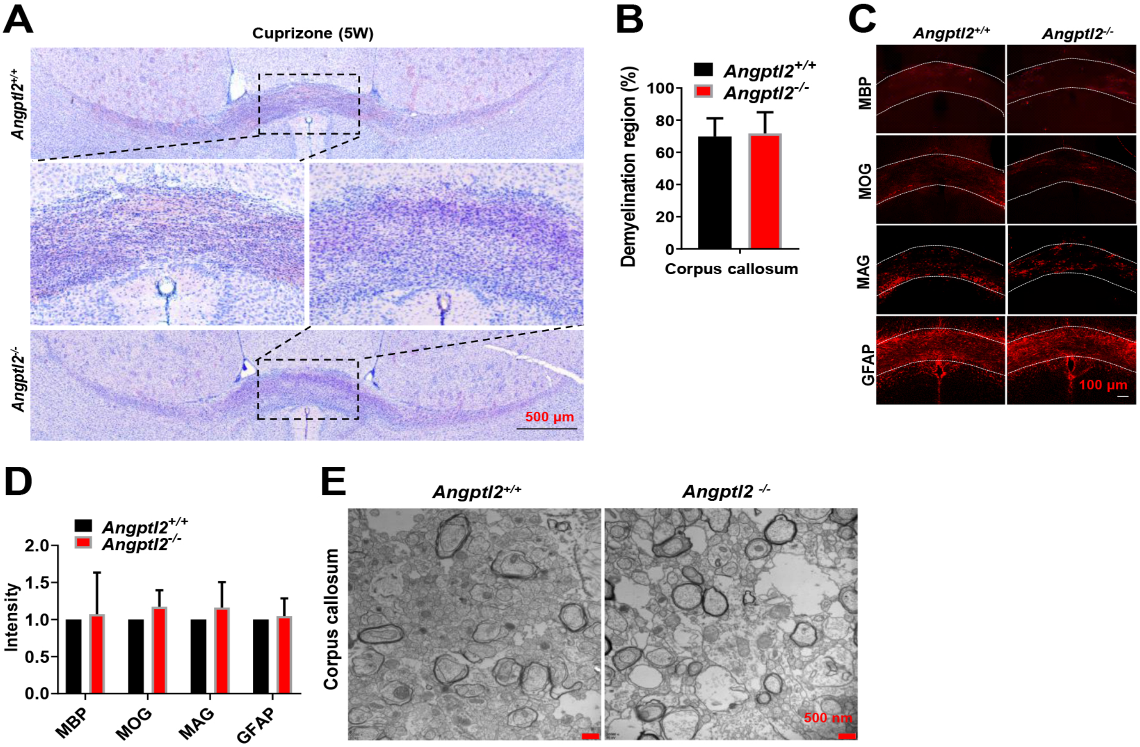
A
Cuprizone (5W)
Angptl2+/+
Angptl2-/-
500 μm
B
Angptl2+/+
Angptl2-/-
Demyelination region (%)
100
80
60
40
20
0
Corpus callosum
C
Angptl2+/+
Angptl2-/-
MBP
MOG
MAG
GFAP
100 μm
D
Angptl2+/+
Angptl2-/-
Intensity
2.0
1.5
1.0
0.5
0.0
MBP
MOG
MAG
GFAP
E
Angptl2+/+
Angptl2 -/-
Corpus callosum
500 nm

Figure S4. related to Figure 4. ANGPTL2 promotes oligodendrocyte differentiation and remyelination under pathological conditions. (A) Representative images of the corpus callosum region stained with oil red O in *Angptl2*$^{+/+}$ and *Angptl2*$^{-/-}$ mice after cuprizone treatment for five weeks. Scale bars, 500 µm. (B) Quantitative data of the demyelinating areas in Panel A; six sections from each mouse were analyzed (n=7). (C) Immunofluorescence staining of MBP, MOG, MAG and GFAP in the corpus callosum region from *Angptl2*$^{+/+}$ and *Angptl2*$^{-/-}$ mice after cuprizone treatment for five weeks. Scale bars, 100 µm. (D) Quantification of the fluorescence intensity of MBP, MOG, MAG and GFAP in the corpus callosum in Panel C. Four sections from each mouse were analyzed (n=5). (E) Representative electron microscopy images of the corpus callosum from *Angptl2*$^{+/+}$ and *Angptl2*$^{-/-}$ mice after cuprizone treatment for 5 weeks (n =3); scale bars, 500 nm.

Figure S5

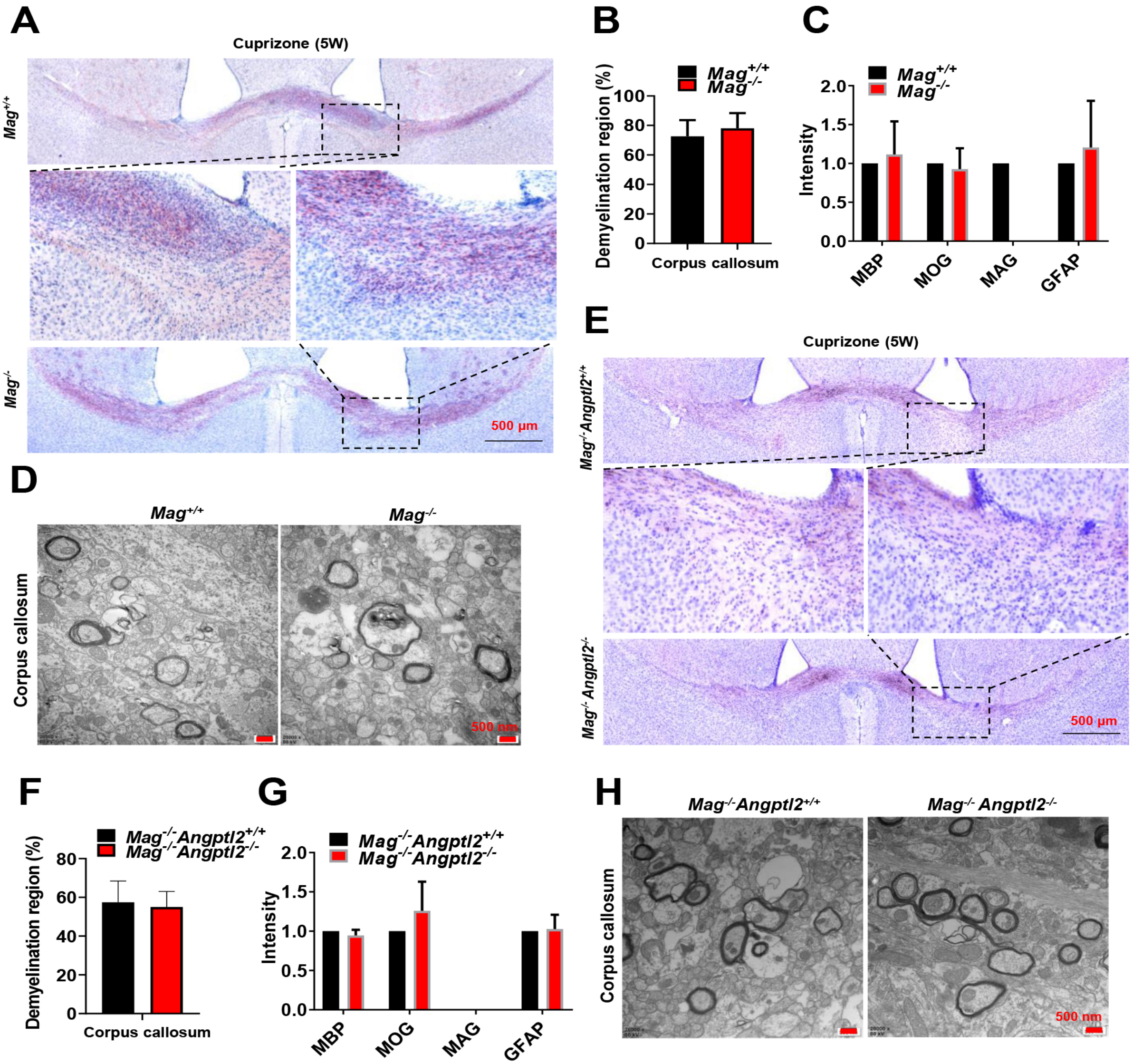
A
Cuprizone (5W)
Mag+/+
Mag-/-
500 μm
B
Mag+/+
Mag-/-
Demyelination region (%)
0
20
40
60
80
100
Corpus callosum
C
Mag+/+
Mag-/-
Intensity
0.0
0.5
1.0
1.5
2.0
MBP
MOG
MAG
GFAP
D
Mag+/+
Mag-/-
Corpus callosum
500 nm
E
Cuprizone (5W)
Mag-/-Angptl2+/+
Mag-/- Angptl2-/-
500 μm
F
Mag-/-Angptl2+/+
Mag-/-Angptl2-/-
Demyelination region (%)
0
20
40
60
80
Corpus callosum
G
Mag-/-Angptl2+/+
Mag-/-Angptl2-/-
Intensity
0.0
0.5
1.0
1.5
2.0
MBP
MOG
MAG
GFAP
H
Mag-/-Angptl2+/+
Mag-/- Angptl2-/-
Corpus callosum
500 nm

Figure S5. related to Figure 5. ANGPTL2 promotes remyelination via its receptor MAG. (A) Representative images of the corpus callosum region stained with oil red O in *Mag*$^{+/+}$ and *Mag* $^{-/-}$ mice after cuprizone treatment for five weeks. Scale bars, 500 μm. (B) Quantification of the demyelinating areas in Panel A; six sections from each mouse were analyzed (n=4). (C) Quantification of the fluorescence intensity of MBP, MOG, MAG and GFAP in the corpus callosum from *Mag*$^{+/+}$ and *Mag* $^{-/-}$ mice after cuprizone treatment for five weeks; four sections from each mouse were analyzed (n=4). (D) Representative electron microscopy images of the corpus callosum from *Mag*$^{+/+}$ and *Mag*$^{-/-}$ mice after cuprizone treatment for five weeks (n=3); scale bars, 500 nm. (E) Representative images of the corpus callosum region stained with oil red O in *Mag*$^{-/-}$*Angptl2*$^{+/+}$ and *Mag*$^{-/-}$*Angptl2*$^{-/-}$ mice after cuprizone treatment for five weeks. Scale bars, 500 μm. (F) Quantification of the demyelinating areas in Panel E; six sections from each mouse were analyzed (n=3). (G) Quantification of the fluorescence intensity of MBP, MOG, MAG and GFAP in the corpus callosum region from *Mag*$^{-/-}$*Angptl2*$^{+/+}$ and *Mag*$^{-/-}$*Angptl2*$^{-/-}$ mice after cuprizone treatment for five weeks; four sections from each mouse were analyzed (n=3). (H) Representative electron microscopy images of the corpus callosum from *Mag*$^{-/-}$*Angptl2*$^{+/+}$ and *Mag*$^{-/-}$*Angptl2*$^{-/-}$ mice after cuprizone treatment for five weeks (n=3); scale bars, 500 nm.

Figure S6

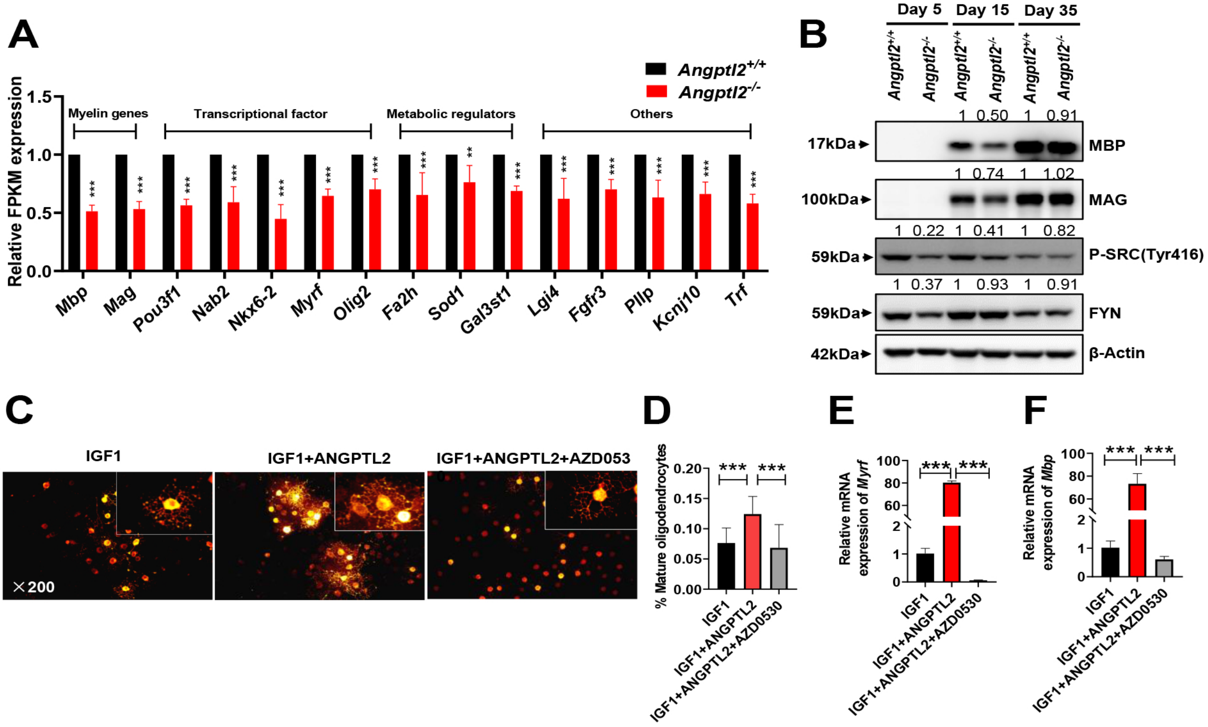
A
Angptl2+/+
Angptl2-/-
Relative FPKM expression
Myelin genes
Transcriptional factor
Metabolic regulators
Others
Mbp
Mag
Pou3f1
Nab2
Nkx6-2
Myrf
Olig2
Fa2h
Sod1
Gal3st1
Lgi4
Fgfr3
Pllp
Kcnj10
Trf
B
Day 5
Day 15
Day 35
17kDa
MBP
100kDa
MAG
59kDa
P-SRC(Tyr416)
59kDa
FYN
42kDa
β-Actin
C
IGF1
IGF1+ANGPTL2
IGF1+ANGPTL2+AZD053
×200
D
% Mature oligodendrocytes
E
Relative mRNA expression of Myrf
F
Relative mRNA expression of Mbp
IGF1
IGF1+ANGPTL2
IGF1+ANGPTL2+AZD0530

Figure S6. related to Figure 6. ANGPTL2-MAG induces Fyn-mediated signaling to enhance the differentiation of oligodendrocytes. (A) The relative FPKM of candidate genes related to the myelin sheath and ensheathment of neurons in the brain tissue of *Angptl2*$^{-/-}$ mice at day 15 as determined by RNA sequencing (n=3). (B) The protein levels of MBP, MAG, the active form FYN (anti-p-SRC Tyr416 antibody) and FYN in the brains of *Angptl2*$^{+/+}$ *and Angptl2*$^{-/-}$ mice were measured by immunoblot analysis. Ratio of MBP/β-actin, MAG/β-actin, active form FYN/β-actin and FYN/β-actin was quantified and normalized against *Angptl2*$^{+/+}$, respectively. One representative experiment is shown. (C) Immunofluorescence staining for MBP in HCN cells 72 hours after induction. (D) Quantitative data in Panel C are shown; A total of 10 sections were counted. (E-F) The mRNA levels of *Myrf* (E) and *Mbp* (F) in HCN cells treated with IGF1, IGF1+ANGPTL2 and IGF1+ANGPTL2+AZD0530, were measured by quantitative RT-PCR (n=3; ***p < 0. 001; one way ANOVA with Dunnett's multiple comparisons test). ANGPTL2-Flag (2μg/ml) and AZD0530 (2μM) were used in panel C-F. One representative experiment is shown. (***p* < 0. 01, ****p* < 0. 001).
