# Supplementary material for: ANGPTL2 binds MAG to efficiently enhance oligodendrocyte differentiation
Source: Cell Biosci. 2023 Feb 28;13:42. doi: 10.1186/s13578-023-00970-3 (PMC9976406; doi:10.1186/s13578-023-00970-3)
Supplement: Supplementary file 2 — Additional file 2: Table S1. Primer sequences for q-RT-PCR and genotyping. [file 13578_2023_970_MOESM2_ESM.docx]

**Additional file 2: Table S1 Primer sequences for q-RT-PCR and genotyping**

| **Primers for q-RT-PCR** | **Sequence (target sequence)** |
| --- | --- |
| Rat *Mag*-F  Rat *Mag*-R  Rat *Mbp*-F  Rat *Mbp* -R | CAGTGACGCCCGAGGACGAT  CACAGTGCGATTCCAGAAGGATTAT  CCAAAGAATAACTGGCAGGGTG  ATCCGTGTCGCTGTGAGGGT |
| Rat *Mog*-F | TGCAGCCAGAGGGCCTTAGCTT |
| Rat *Mog*-R | ATTCAGGCGCTTGCTCTGCGT |
| Rat *PirB*-F | AGGTGGACGGTCAGGCTACT |
| Rat *PirB* -R | TTCTCACTTCACTTGATTATTGGG |
| Rat *Angptl2*-F | TGGAGCCAGAAAGCGAGTA |
| Rat *Angptl2*-R | ACCCCATTGAGGTTGGAGTG |
| Rat*β-Actin*-F | GTAAAGACCTCTATGCCAACA |
| Rat*β-Actin*-R | CTCAGTAACAGTCCGCCTA |
| Mouse *Mbp*-F | CCAAGTTCACCCCTACTCCA |
| Mouse *Mbp*-R | TAAGTCCCCGTTTCCTGTTG |
| Mouse *Mag*-F | AACCAGTATGGCCAGAGAGC |
| Mouse *Mag*-R | GTTCCGGGTTGGATTTTACC |
| Mouse *Pou3f1*-F | AGCACTCGGACGAGGATG |
| Mouse *Pou3f1*-R | TTGAACTGCTTGGCGAACT |
| Mouse *Nab2*-F | TGGCAGAGGGGATAACACAC |
| Mouse *Nab2*-R | CGTTGCAGGACCCGATACAG |
| Mouse *Nkx6-2*-F | AAGTCTGCCCCGTCTCAAC |
| Mouse *Nkx6-2*-R | GGTCTGCTCGAAAGTCTTCTC |
| Mouse *Myrf*-F | GCATGGGCACCGCCCCTAAG |
| Mouse *Myrf*-R | GGGGCGAGTCTGGCAGTGTG |
| Mouse *Oligo2*-F | ATATGGGAACCGAAGCAATG |
| Mouse *Oligo2*-R | CAGGAAGTTCCAGGGATGAA |
| Mouse *Fa2h*-F | CCACTTGGGGGAGAAGTATGA |
| Mouse *Fa2h*-R | TGGGGACACTATACCAGACAG |
| Mouse *Sod1*-F | AACCAGTTGTGTTGTCAGGAC |
| Mouse *Sod1*-R | CCACCATGTTTCTTAGAGTGAGG |
| Mouse *Gal3st1*-F | CAAACAAGCACTTGCAGGAA |
| Mouse *Gal3st1* –R | TGGGGTACTTCTGCTCCATC |
| Mouse *Lgi4*-F | CCTCCGTGGTAACCCGTTC |
| Mouse *Lgi4*-R | GGTGATTCAGTTGTATCTGAGCA |
| Mouse *Fgfr3*-F | GGAGGACGTGGCTGAAGAC |
| Mouse *Fgfr3*-R | GGAGCTTGATGCCCCCAAT |
| Mouse *Pllp*-F | TGATTGCTGACACCCCATACC |
| Mouse *Pllp* -R | GCAGTAATGTAGAGAACCGTGG |
| Mouse *Kcnj10*-F | CTGCCCCGCGATTTATCAGA |
| Mouse *Kcnj10*-R | CATTCTCACATTGCTCCGGC |
| Mouse *Trf*-F | GCAGCCCAAATGACCCAGT |
| Mouse *Trf*-R | GGAGAGGGACCGATCAAAGAT |
| Mouse*β-Actin*-F | GGCTGTATTCCCCTCCATCG |
| Mouse*β-Actin*-R | CCAGTTGGTAACAATGCCATGT |
| **Primers for genotyping** | **Sequences** |
| *Angptl2*-loxp-tF | ATCCTAATGTCCCTCTTGGC |
| *Angptl2*-FRT-tR | CAGGCTGTGAACAGGTTAGTCATC |
| *Mag-com-*F | AAGATGATATTCCTCGCCACC |
| *Mag-*WT*-*R | CGGAAATAGTATTTGCCTCCC |
| *Mag-*Mutant*-*R | TGGATGTGGAATGTGTGCGAG |
