# Supplementary material for: ANGPTL2 binds MAG to efficiently enhance oligodendrocyte differentiation
Source: Cell Biosci. 2023 Feb 28;13:42. doi: 10.1186/s13578-023-00970-3 (PMC9976406; doi:10.1186/s13578-023-00970-3)

Full unedited gel for Figure 1C

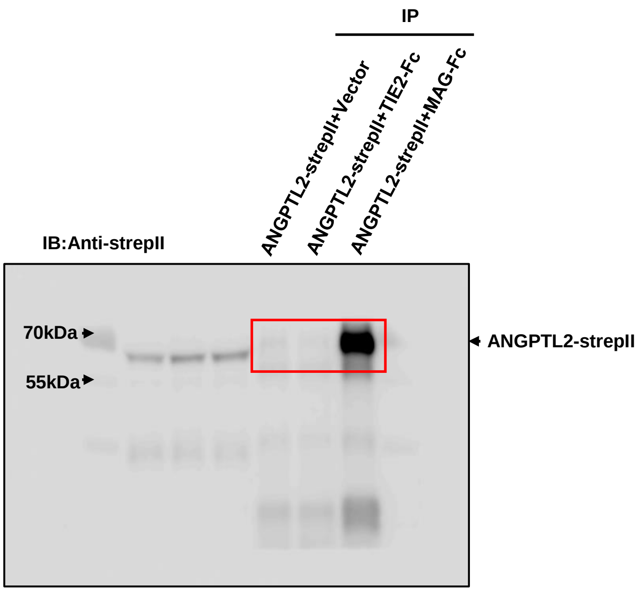

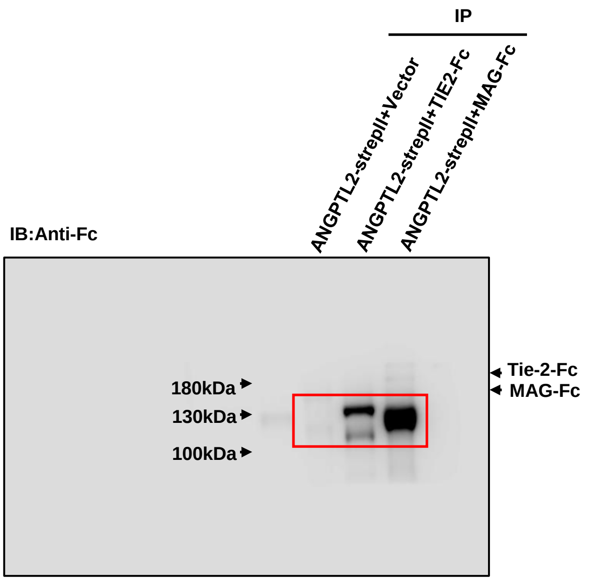

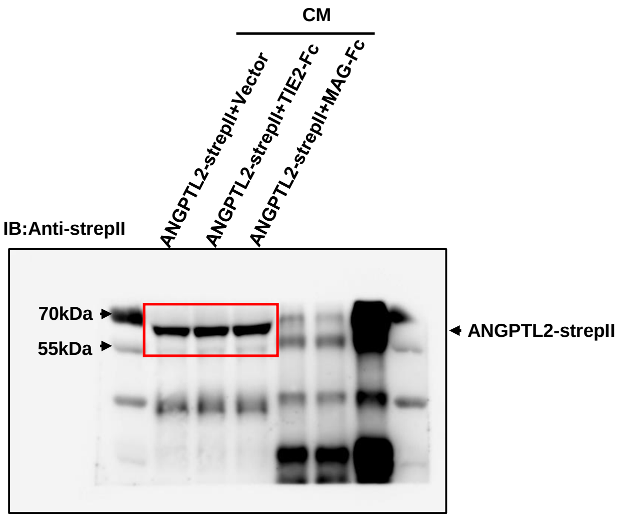

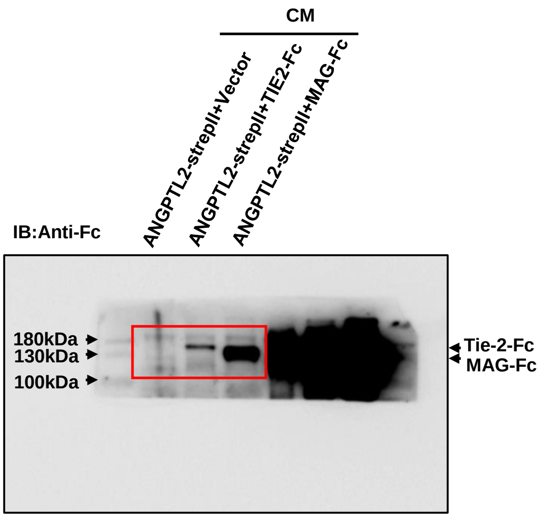

Full unedited gel for Figure S1B

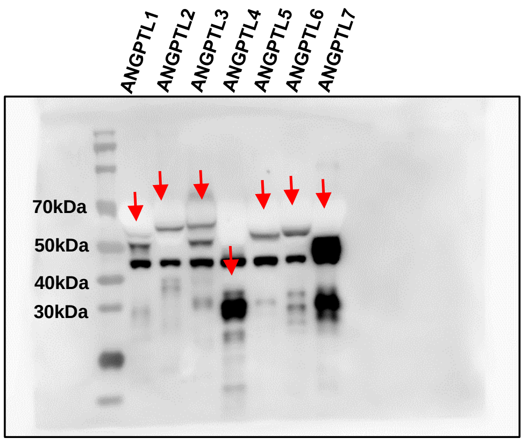

Full unedited gel for Figure 2C

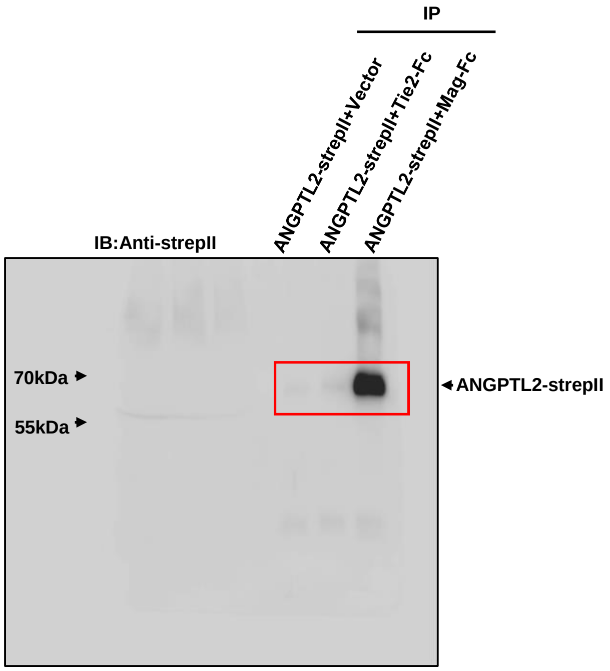

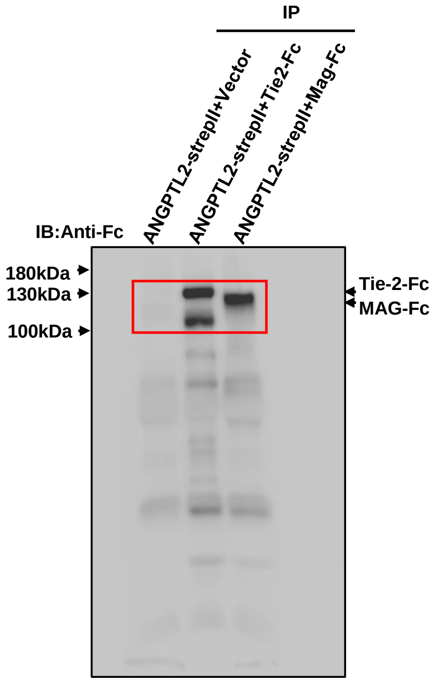

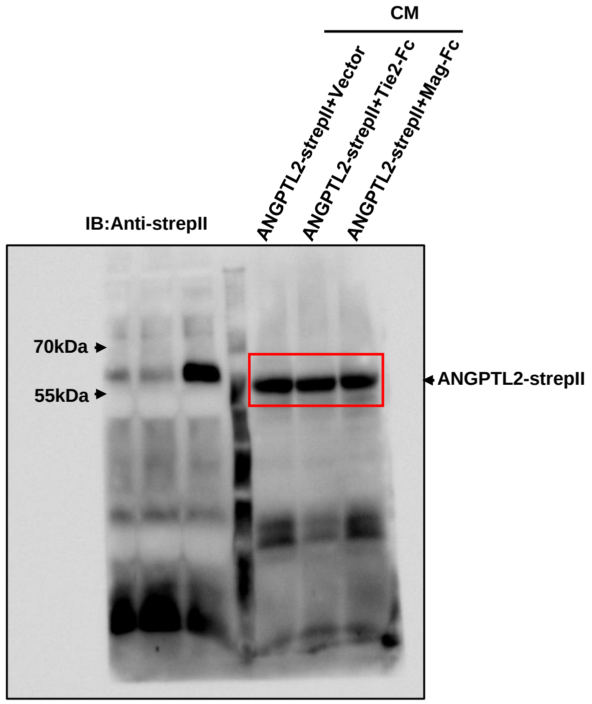

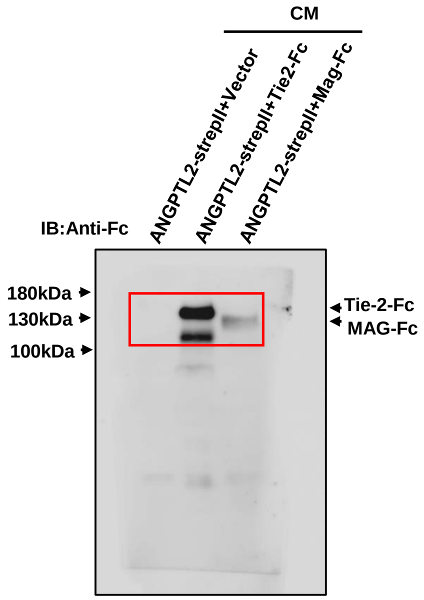

Full unedited gel for Figure S3C

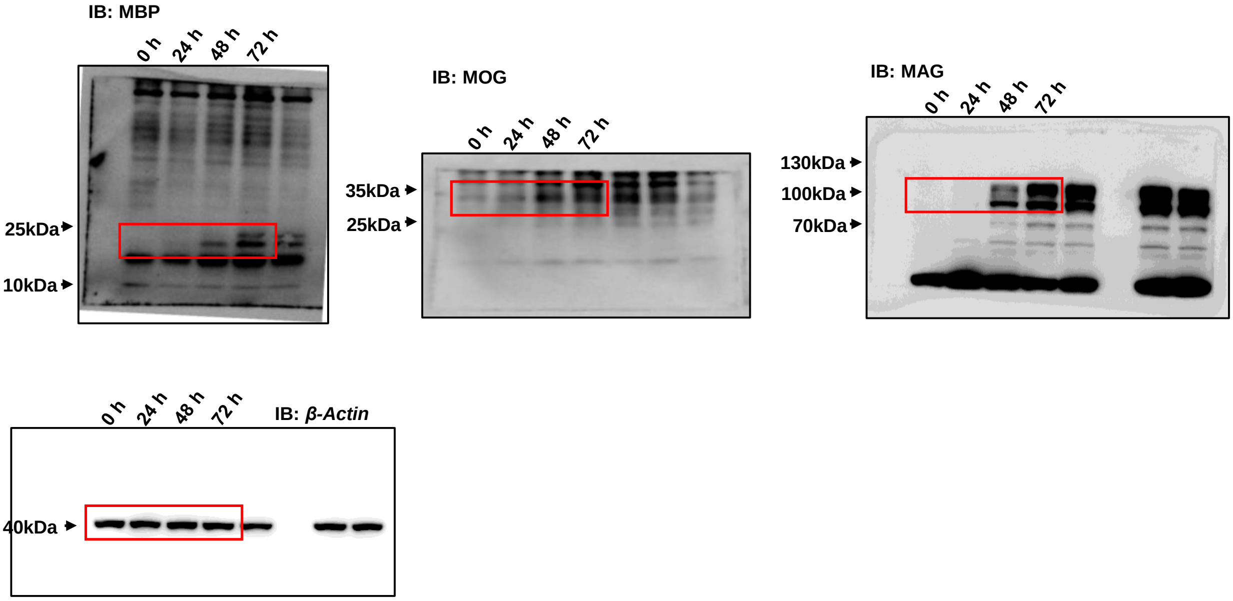

Full unedited gel for Figure S3D

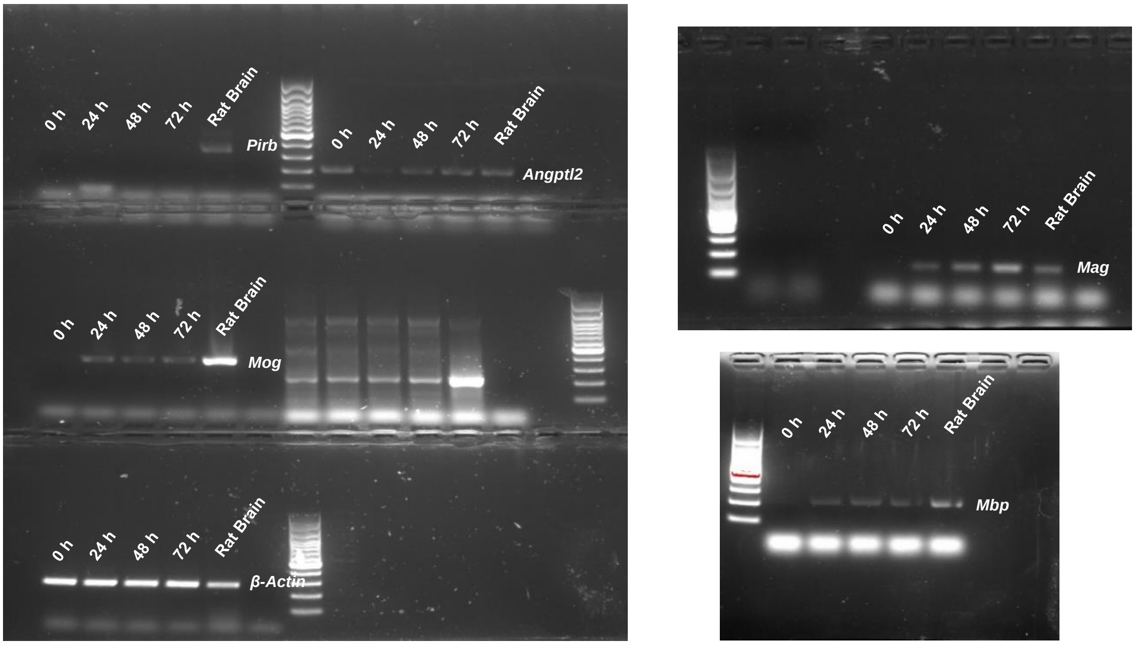

Full unedited gel for Figure S3E

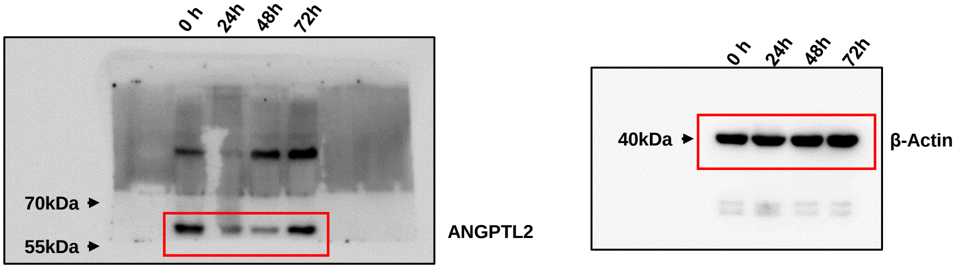

Full unedited gel for Figure S3J

IB: ANGPTL2

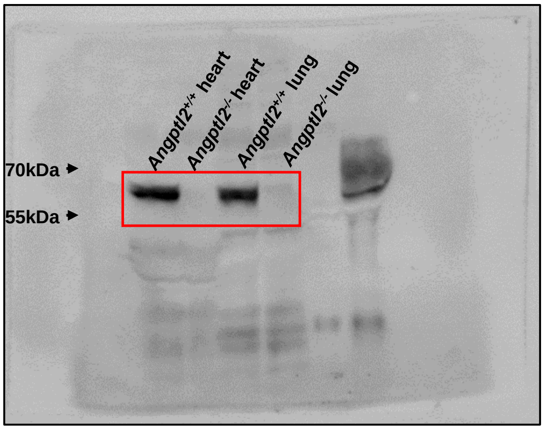

IB:GAPDH

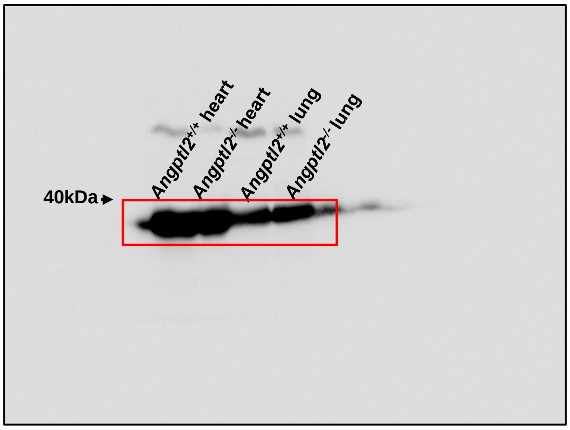

IB: ANGPTL2

IB: ANGPTL2

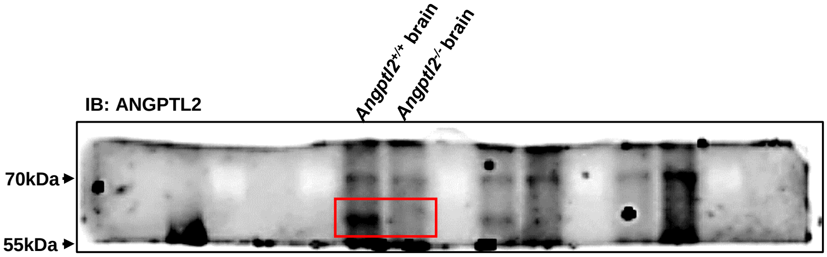

IB:GAPDH

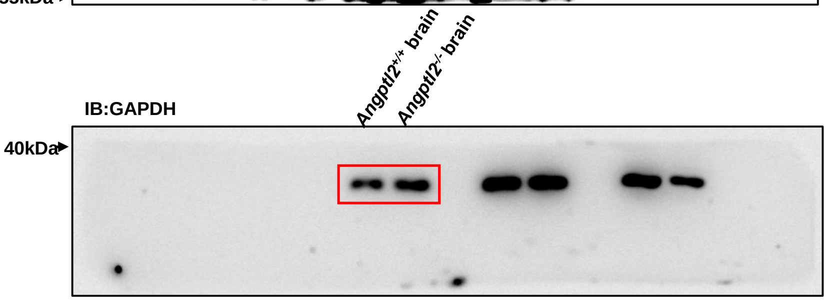

Full unedited gel for Figure 6D

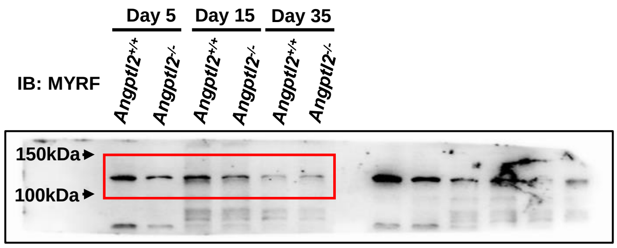

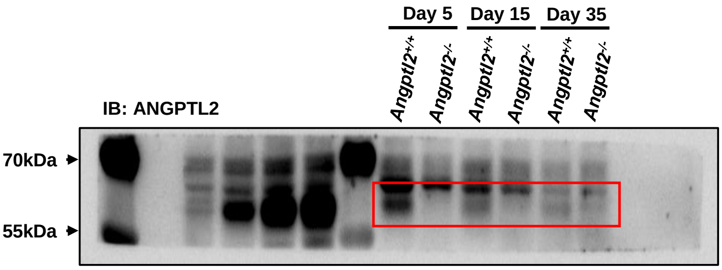

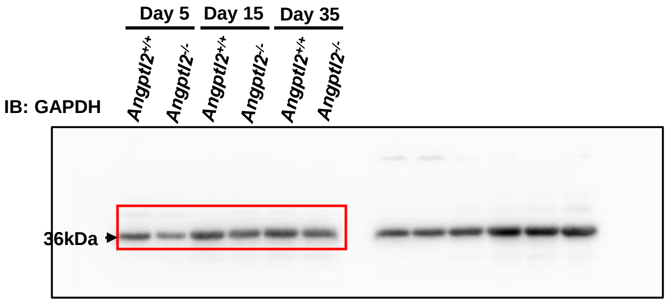

Full unedited gel for Figure 6E

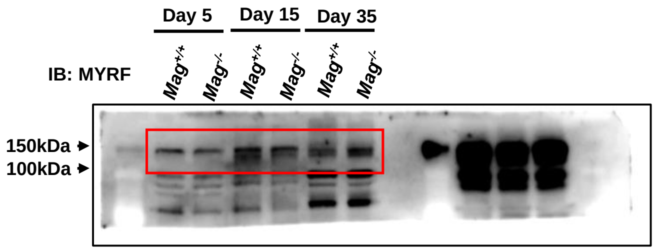

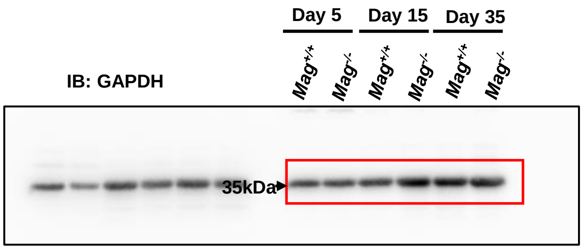

Full unedited gel for Figure 6F

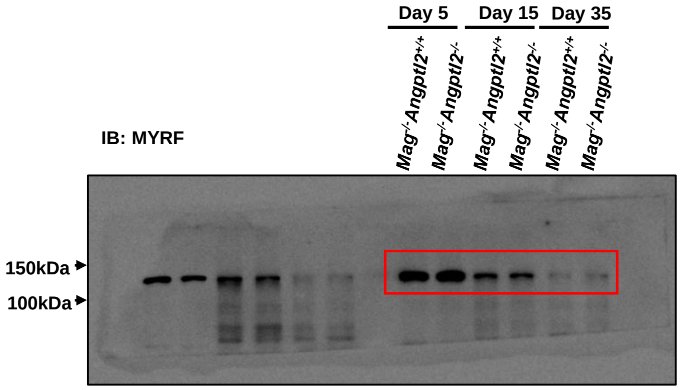

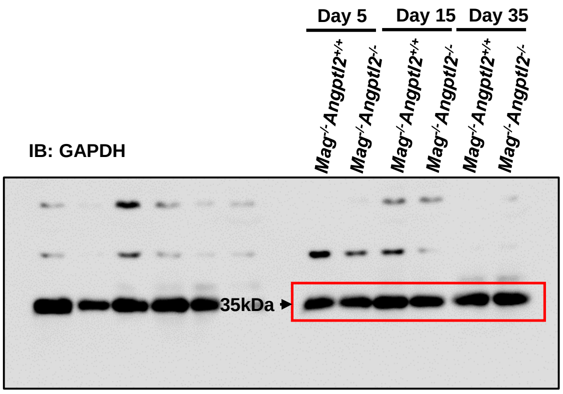

Full unedited gel for Figure 6G

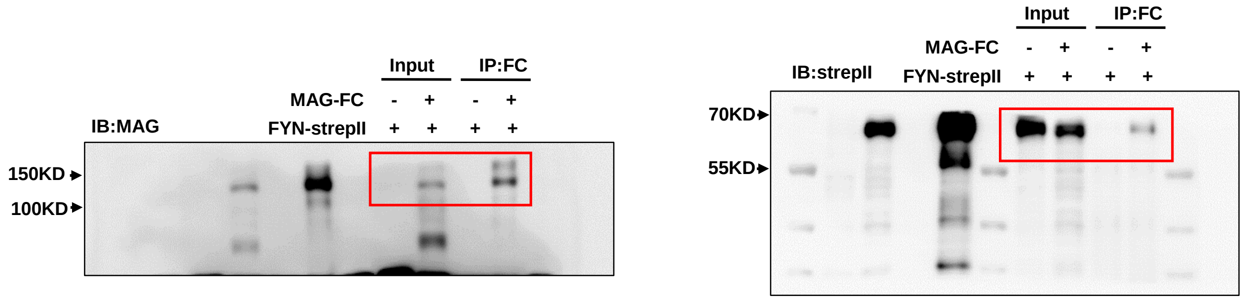

Full unedited gel for Figure 6H

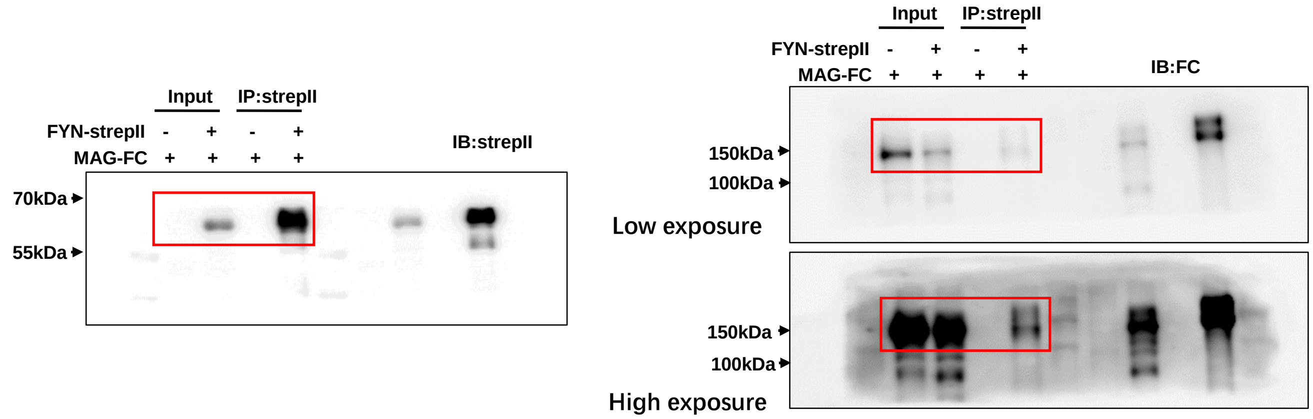

Full unedited gel for Figure 6I

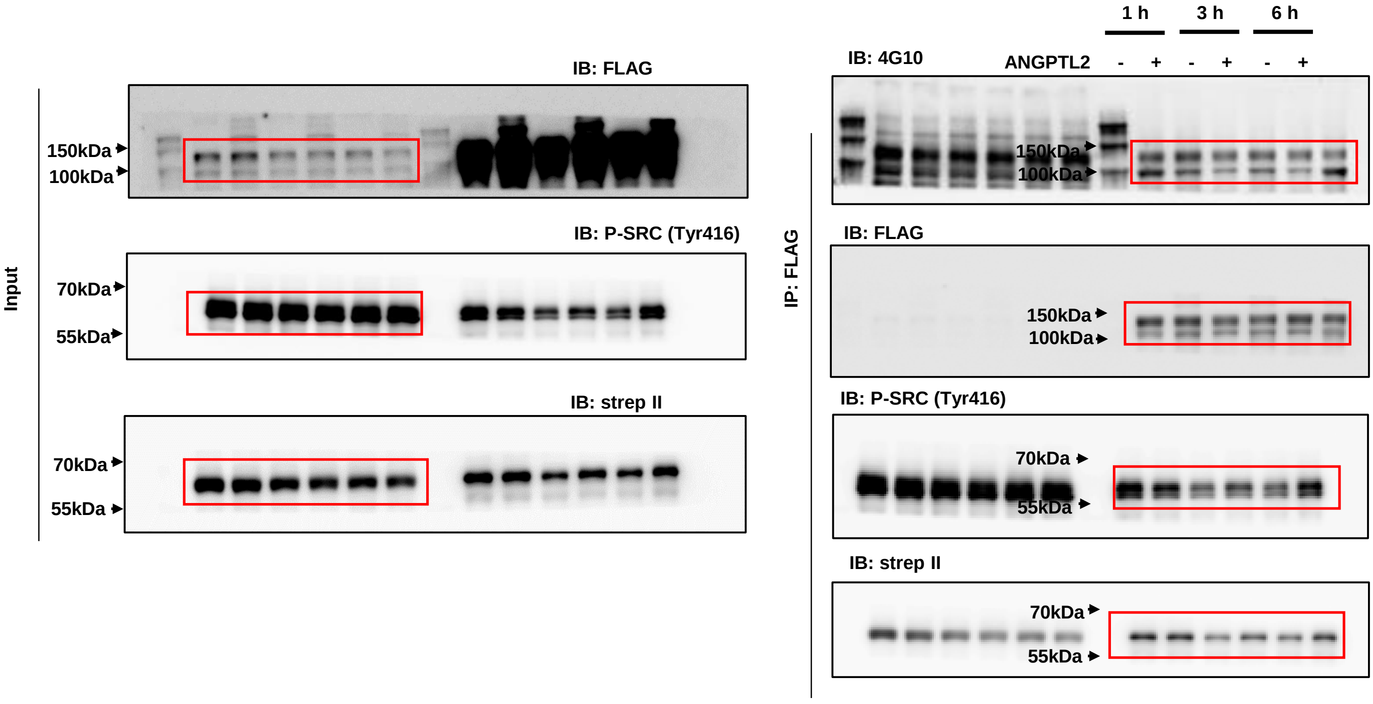

Full unedited gel for Figure 6J

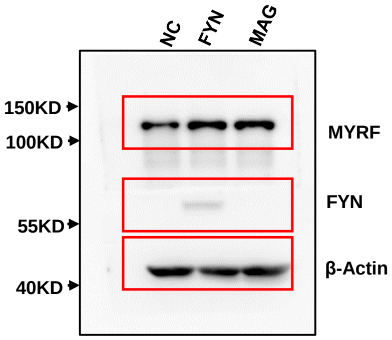

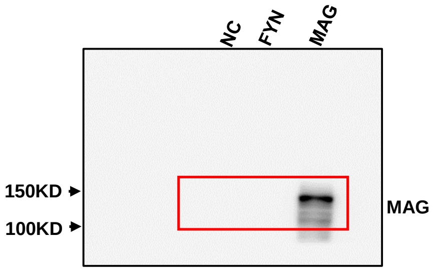

Full unedited gel for Figure 6K

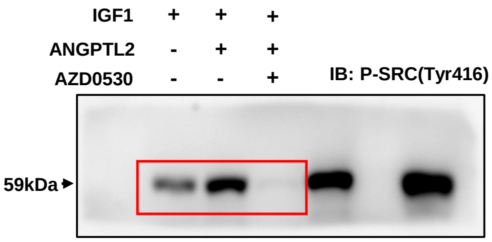

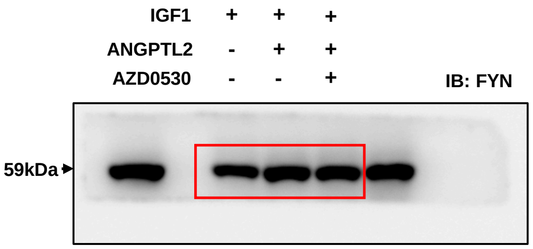

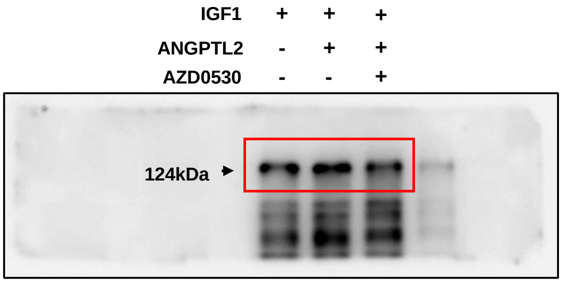

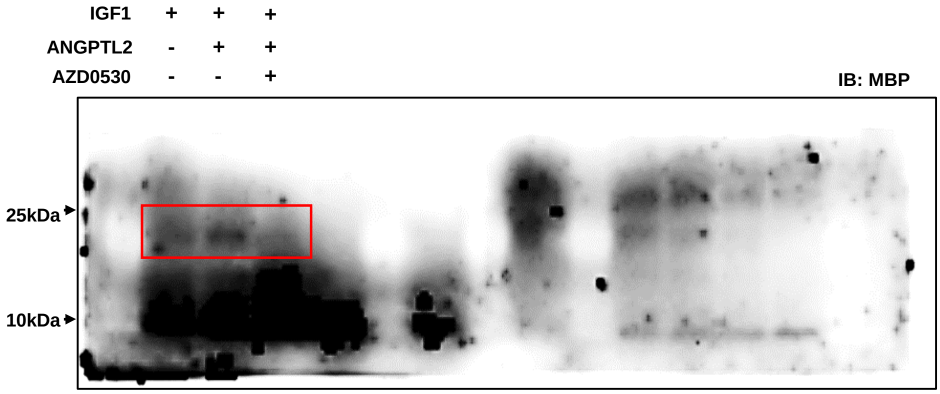

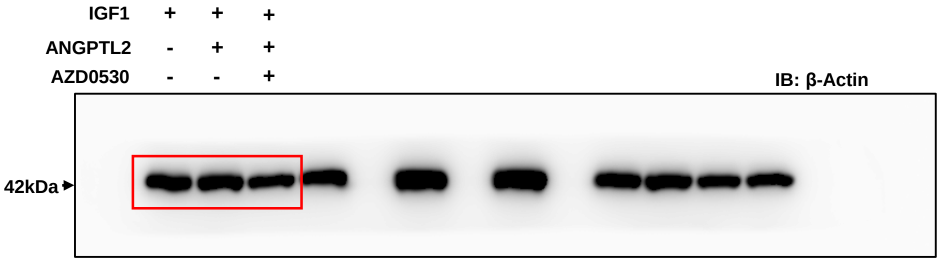

Full unedited gel for Figure S6B

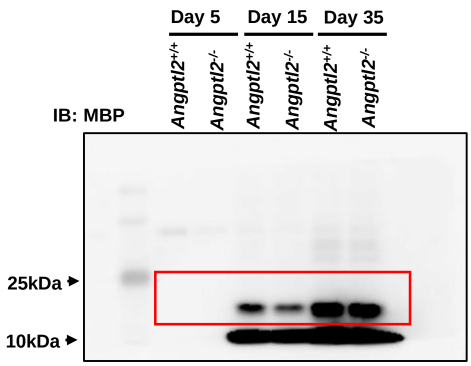

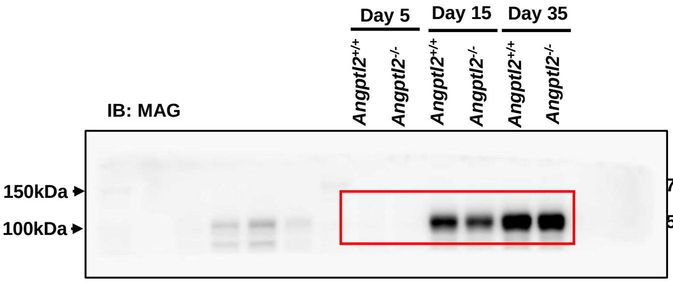

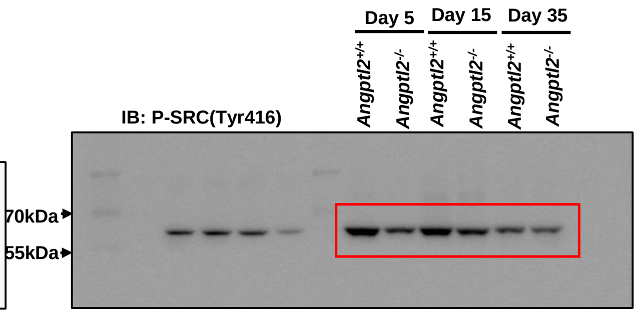

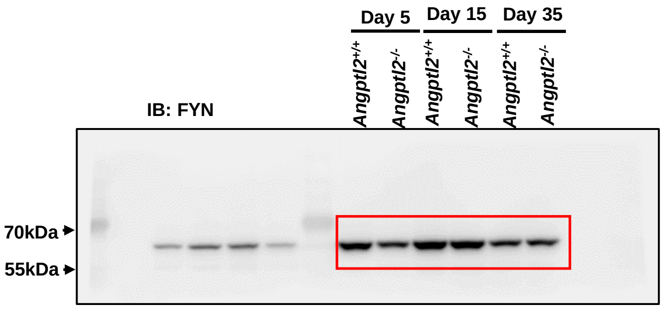

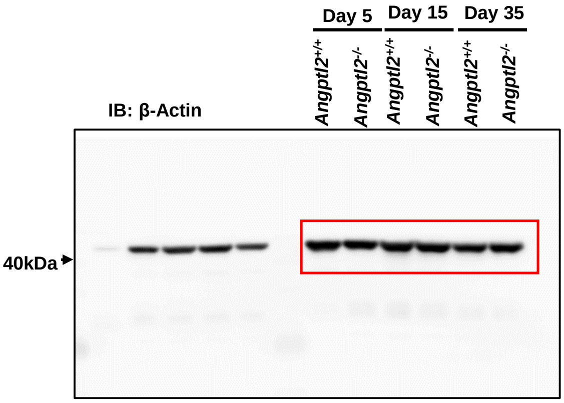

Supplement: Supplementary file 3 — Additional file 3: Unedited images from gels and western blots. [file 13578_2023_970_MOESM3_ESM.pdf]
